# Construction of AIE active fluorescent sensor for highly specific sensing of hydrogen peroxide through turn on fluorescence emission

**DOI:** 10.1039/d6ra02128j

**Published:** 2026-07-02

**Authors:** Iqra Mustafa, Muqadas, Alam Shabbir, Mohammed A. Assiri, Umar Farooq, Sohail Anjum Shahzad

**Affiliations:** a Department of Chemistry, COMSATS University Islamabad, Abbottabad Campus University Road Abbottabad 22060 Pakistan umarf@cuiatd.edu.pk sashahzad@cuiatd.edu.pk; b Department of Chemistry, Faculty of Science, King Khalid University P. O. Box 9004 Abha 61413 Saudi Arabia; c Central Labs, King Khalid University AlQura'a, P. O. Box 960 Abha 61413 Saudi Arabia

## Abstract

Hydrogen peroxide (H_2_O_2_) is a poisonous chemical commonly used as a crucial component in the production of peroxide-based explosives and plays an important role in various biological processes. Thus, it is crucial to detect H_2_O_2_ vapor and solutions for safety and diagnostic purposes. Proposed sensor MPT exhibited remarkable photophysical characteristics regarding the structure–property relationship, such as solvatochromism and aggregation-induced emission (AIE) based on J-aggregates. Following this, a carefully designed sensor MPT was employed for turn-on fluorescence sensing of H_2_O_2_*via* strong hydrogen bonding, achieving limits of detection as low as 80.6 nM. This distinct sensing phenomenon increases the versatility and potential applications of our sensor for selective and sensitive H_2_O_2_ detection. ^1^H NMR, DLS, FTIR, mass spectrometry (LC-MS), titration experiments, and detailed density functional theory calculations elucidated the unique sensing mechanism. Due to effective interactions, the sensor detects H_2_O_2_ with key attributes such as exceptional selectivity, nanoscale detection, rapid response, and outstanding photostability. Additionally, sensor MPT-embedded fluorescent strips were successfully used as a portable device for the rapid detection of H_2_O_2_ vapor. Furthermore, sensor MPT was also able to monitor H_2_O_2_ in industrial water. Moreover, a logic-gate model was fabricated to detect H_2_O_2_ in real time. The sensor MPT was employed to measure concentration of hydrogen peroxide in commercially available products such as hair bleach, mouthwash, and disinfectant. Consequently, our sensor offers promising characteristics for trace detection of H_2_O_2_ in real samples, making it highly valuable.

## Abstract 2

Hydrogen peroxide (H_2_O_2_) is a poisonous chemical commonly used as a crucial component in the production of peroxide-based explosives and plays an important role in various biological processes.

## Graphical abstract

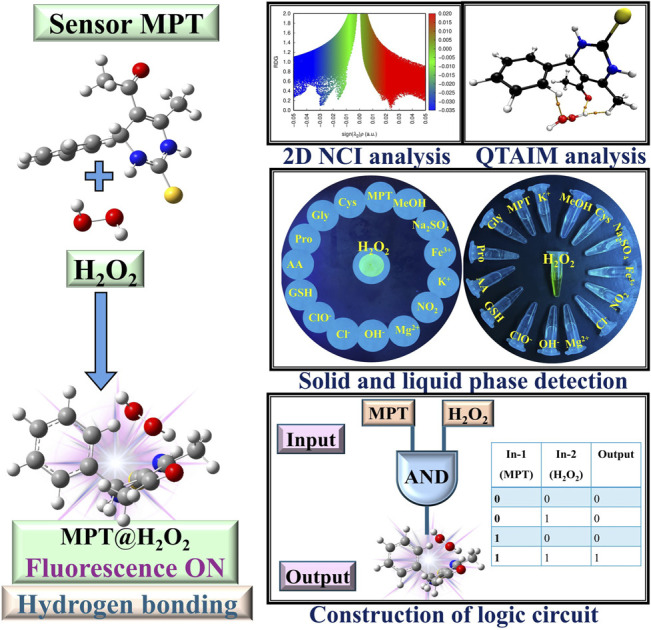

## 1. Introduction

Hydrogen peroxide (H_2_O_2_) is a well-known hazardous chemical and a potential threat agent.^1^ H_2_O_2_ is an inorganic, volatile compound and powerful oxidant that plays a crucial role in various chemical, industrial, medical, environmental, and biological processes.^2,3^ It is widely utilized in industrial applications and in food processing as a bleaching agent, wastewater treatment processes as an effective oxidizing agent, and various chemical syntheses.^4^ It is also extensively used in the bleaching of paper pulp, textiles, and hair, while its strong antimicrobial properties make it an effective disinfectant. Additionally, H_2_O_2_ is employed as a precursor for easily synthesized peroxide-based explosives, including hexamethylene triperoxide diamine (HMTD), which are frequently exploited in various terrorist activities.^5^ Biologically, H_2_O_2_ plays a critical role in numerous physiological processes, including host defense mechanisms, oxidative biosynthetic pathways, metabolic regulation, and cellular signaling.^6,7^ H_2_O_2_ is a significant product of various highly selective oxidases, making it an essential mediator in numerous biological processes. Given its significance in both environmental and biological contexts, H_2_O_2_ is present in various ecosystems, human biological fluids, and food products. The concentration of H_2_O_2_ in surface waters such as oceans, rain, snow, rivers, and lakes is a key factor affecting the health of aquatic flora and fauna, as well as the overall quality of air and water.^8–12^ In clinical settings, measuring the levels of H_2_O_2_ in biological fluids such as urine and blood plasma can provide valuable insights into metabolic disorders, including those associated with diabetes and pulmonary diseases.^13–16^ In the context of food and beverages, H_2_O_2_ residues can persist in final products after processes such as pasteurization, sterilization, and packaging. Food safety guidelines from Food Standards Australia and New Zealand stipulate that H_2_O_2_ concentrations must remain below 147 µM in these finished products.^17^ Exceeding this threshold can pose serious health risks as elevated levels of H_2_O_2_ are deemed hazardous for human consumption, particularly when ingested or inhaled.

The widespread use and inherent chemical instability of H_2_O_2_ elevate the presumptive risk of public exposure during its storage and transport. Elevated oxidative stress due to H_2_O_2_ exposure has been linked to significant DNA damage and serves as a biomarker for a range of pathological conditions, such as Parkinson's disease, Alzheimer's disease, strokes, atherosclerosis, and certain cancers.^18,19^ Elevated or abnormal concentrations of H_2_O_2_ in the human body can lead to tissue damage and tumorigenesis. Thus, implementing reliable monitoring techniques for H_2_O_2_ in both liquid and vapor forms is crucial to maintain its concentrations within safe and manageable limits for protecting health, safety and environmental integrity.

Researchers have explored a variety of analytical methods for detecting H_2_O_2_ including electrochemical techniques,^20^ colorimetry,^21^ electroanalysis,^22^ high performance liquid chromatography^23^ and fluorescence spectroscopy.^24^ Among these, fluorescence spectroscopy offers distinct advantages, such as straightforward operational procedure, cost effectiveness and enhanced selectivity and sensitivity.^25^ This technique is particularly notable for its rapid response times, superior sensitivity, portability and user-friendly system operations. Various synthetic, nanoparticle-based and organic small molecule fluorescent probe^26^ and various other fluorescent probes^27^ have been developed for the detection of H_2_O_2_. Researchers have focused on synthetic probes because they are less toxic than traditional methods. Fluorescent probes for H_2_O_2_ detection are typically based on diverse reaction mechanisms, including deprotonation-type reactions, boronic ester oxidation, Baeyer–Villiger type reactions and other oxidative transformations.^3,28,29^ Although these probes demonstrate commendable selectivity for H_2_O_2_ among related analytes, reaction-based probes have significant drawbacks. These include a delayed response time, relatively elevated limit of detection (LOD), aggregation-caused quenching (ACQ),^30^ irreversibility and the potential generation of toxic byproducts.^31^ To address these limitations, there is a compelling need to develop probes that use non-covalent interactions to detect H_2_O_2_ because non-covalent interactions are weak and dynamic, making sensors capable of detecting very small amounts of analytes, producing a measurable signal.

In this perspective, structurally diverse pyrimidine derivative 1-(6-methyl-4-phenyl-2-thioxo-1,2,3,4-tetrahydropyrimidin-5-yl)ethan-1-one (MPT) has been developed through Biginelli reaction to explore its photophysical properties. As predicted, the synthesized compound exhibits aggregation-induced emission (AIE) characteristics and a red shift in its emission.^32–35^ This AIE sensor has high specificity and selectivity towards H_2_O_2_ due to its distinct geometry. MPT has rigid and conjugated heterocyclic framework consisting of phenyl ring attached to a pyrimidine core. The structural arrangement provides a well-defined spatial orientation of functional groups including the carbonyl, thione and amine moieties. In the presence of H_2_O_2_, the fluorescence emission from the sensor MPT is significantly enhanced due to hydrogen bonding between the functional groups of the sensor and H_2_O_2_. Hydrogen bonding between H_2_O_2_ and the sensor's terminal oxygen atom limits intramolecular vibrations, leading to enhanced emissions. Comprehensive DFT investigations were also conducted to gain a deep understanding of the interaction between the sensor MPT and H_2_O_2_. Additionally, the sensor's selectivity, stability and repeatability have been evaluated. Notably, sensor MPT showed exceptional capability for detecting H_2_O_2_ vapors. Apart from this, sensor MPT was effectively utilized for sensing H_2_O_2_ in real samples, making it highly practical for applications.

## 2. Methodology

### 2.1 Synthesis

The desired compound was synthesized by the procedure reported in literature.^36^ Benzaldehyde (4.0 mmol), thiourea (4.5 mmol), ethyl acetoacetate (2.5 mmol), and triphenylphosphine (0.4 mmol) were taken in a round-bottom flask and heated at 110 °C for 13 h. After completion of the reaction, the reaction mixture was added to crushed ice with constant stirring. Filter the product, then wash it in cold water and dry it. Recrystallisation was done with 95% ethanol to obtain a pure product MPT as pale yellow color (70% yields). ^1^H-NMR (400 MHz, DMSO-*d*_6_), (*δ* = ppm): 10.27 (s, 1H, NH), 9.74 (d, 1H, *J* = 1.6 Hz, NH), 7.34 (t, 2H, *J* = 7.1 Hz, ArH), 7.27 (m, 1H, ArH), 7.22 (d, 2H, *J* = 7.2 Hz, ArH), 5.29 (d, 1H, *J* = 3.3 Hz, CH), 2.33 (s, 3H, CH_3_), 2.15 (s, 3H, CH_3_).^37^^13^C-NMR (100 MHz, DMSO-*d*_6_), (*δ* = ppm): 195.2 (C=O), 174.5 (C=S), 145.0 (1 × C), 143.3 (1 × C), 129.1 (2 × CH), 128.1 (1 × CH), 127.0 (2 × CH), 110.9 (1 × C), 54.2 (CH), 30.9 (*CH*_3_–CO), 18.7 (CH_3_). DEPT-135 NMR (100 MHz, DMSO-*d*_6_), (*δ* = ppm): 18.7 (*CH*_3_), 30.8 (*CH*_3_–CO), 54.2 (CH), 127.0 (2 × CH), 128.1 (1 × CH), 129.1 (2 × CH). LRMS (ESI): *m*/*z* [M − H]^−^ calculated for C_13_H_14_N_2_OS, 245.33; found, 245.33 (Fig. S1).

### 2.2 UV-visible and fluorescence experiments

To determine the optimal concentration of the sensor MPT, we conducted a series of UV-visible and fluorescence experiments across various concentrations (5–50 µM) in pure DMF solutions. A concentration of 30 µM was selected based on observed absorption and maximum emission intensity. In our subsequent emission studies, we tested different water fractions ranging from 10% to 90% within a binary solvent system, and a 50% water fraction was selected. This optimized concentration was subsequently employed in titration experiments. To evaluate the selectivity of the sensor MPT, 1 mM stock solutions of various analytes, including H_2_O_2_, Na_2_SO_4_, NO_2_, K^+^, Mg^2+^, Fe^3+^, Cl^−^, OH^−^, ClO^−^, HCHO (formalin, 37%), N_2_H_4_, methanol (CH_3_OH), cysteine (Cys), proline (Pro), glutathione (GSH), glycine (Gly), and ascorbic acid (AA) were scanned. The spectral data were assessed by systematically varying H_2_O_2_ concentrations relative to the other analytes, using a quartz cuvette. Emission experiments were conducted at an excitation wavelength of 290 nm. All experiments were carried out at an ambient temperature.

### 2.3 DFT methodology

Theoretical DFT/TD-DFT calculations were performed using Gaussian 09 software.^38^ GaussView 5.0, GaussSum, Multiwfn 3.7 and VMD software were used for visualization of the results.^39^ Geometric optimization and sensor MPT calculations of interaction energies between the sensor MPT and the analyte are the main purposes of DFT/TD-DFT calculations. All-time dependent density functional theory (TD-DFT) was performed on functional B3LYP and ωB97XD, along with basis set 6-311G**.^40,41^ They were used for optimization and interaction energy calculations.

The electronic properties were examined by accomplishment of frontier molecular orbital (FMO), density of state (DOS) and natural bond orbital (NBO) studies. Natural bond orbital analysis specifies the charge transfer between sensor MPT and analyte (H_2_O_2_). To evaluate the presence of van der Waals interactions among the two interacting species (sensor MPT and analyte), non-covalent interaction (3D isosurface and 2D RDG) analysis was performed using Multiwfn and VMD software.^42^ NCI analysis was carried out to get the 3D isosurface and 2D reduced density gradient plot. Bader's QTAIM analysis was performed to study the intermolecular interactions.^43^ In QTAIM, the bond critical points are investigated by various topological parameters to describe the nature and type of interactions between sensor MPT and H_2_O_2_.

## 3. Results and discussion

### 3.1 Chemistry

**Scheme 1:**
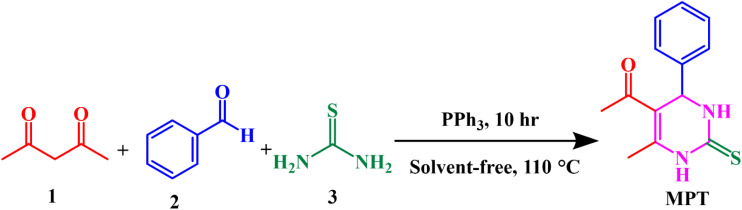
Synthetic method for obtaining dihydropyrimidine based sensor MPT.

The dihydropyrimidine based sensor MPT was synthesized as shown in Scheme 1, which involves formation of acylinium by reaction of benzaldehyde and thiourea. Later addition of enolate and cyclodehydration results in formation of desired product MPT. The compound MPT was characterized by NMR spectroscopy and mass spectrometry. The detailed NMR spectra of MPT are cited in the SI (SI-2).

### 3.2 Optimization

Concentrations of sensor MPT ranging from 5 to 50 µM in pure DMF were examined to evaluate its fluorescence emission response (*λ*_ex_ = 290 nm). The inner filter effect (IFE) significantly influenced the emission characteristics of fluorescent molecules. As the concentration of sensor MPT in solutions increased, resulting in a decrease in emission intensity due to primary inner filter effect. To mitigate the primary IFE, dilution of the solution was necessary. The sensor MPT was excited at a wavelength of 290 nm, where it exhibited maximum emission intensity (*λ*_em_ = 356 nm) at a concentration of 30 µM in pure DMF (Fig. S2a). Therefore, this concentration was chosen for subsequent optical studies.

Additionally, UV visible spectra were obtained at concentrations from 5 to 50 µM, and 30 µM was selected as the optimized concentration (Fig. S2b). Notably, the primary IFE was minimal at 30 µM, the negligible overlap between the emission and absorption spectra of MPT exclude the presence of secondary inner filter effect (IFE), along with a Stokes shift of 35 nm (Fig. S2c).

### 3.3 Solvatochromism

Emission spectra of the MPT sensor were recorded in various solvent polarities, ranging from non-polar to polar solvents (protic and aprotic) such as ethyl acetate (EA), ethanol (CH_3_CH_2_OH), *N*-dimethylformamide (DMF), *n*-hexane (HEX), methanol (CH_3_OH), tetrahydrofuran (THF) and dichloromethane (DCM) (Fig. S3a). The observed red shift indicates a longer wavelength in more polar solvents, exemplifies the concept of positive solvatochromism.^44^ This phenomenon occurs when the excited state of a molecule is more favorably stabilized by polar solvents compared to its ground state. Ground state (S_0_) and excited state (S_1_) exhibit different dipole moments. When the excited state possesses a higher dipole-moment due to its increased polarity, polar solvents can stabilize it more effectively than the ground state. As a result, the energy gap between S_1_ and S_0_ decreases, leading to red-shifted emissions (longer wavelength). For instance, in the case of hexane, a non-polar solvent, stabilizes neither state resulting in large energy gap and consequently a short wavelength emission at 315 nm.

Transitioning to polar aprotic solvents like DCM, THF, EA and DMF, we observed a moderate stabilization of the S_1_ state, giving rise to an intermediate emission shift to 353 nm, 354 nm, 355 nm, 357 nm. In contrast, polar protic solvents such as methanol and ethanol strongly stabilize the S_1_ state, leading to the smallest energy gap and the longest wavelength emission at 365 nm. This illustrates the varying effects of solvent polarity on the electronic states of molecules and their corresponding emissions. Therefore, DMF was chosen as a suitable solvent for additional studies due to its non-volatility.

### 3.4 Aggregation-induced emission (AIE) behavior

**Fig. 1:**
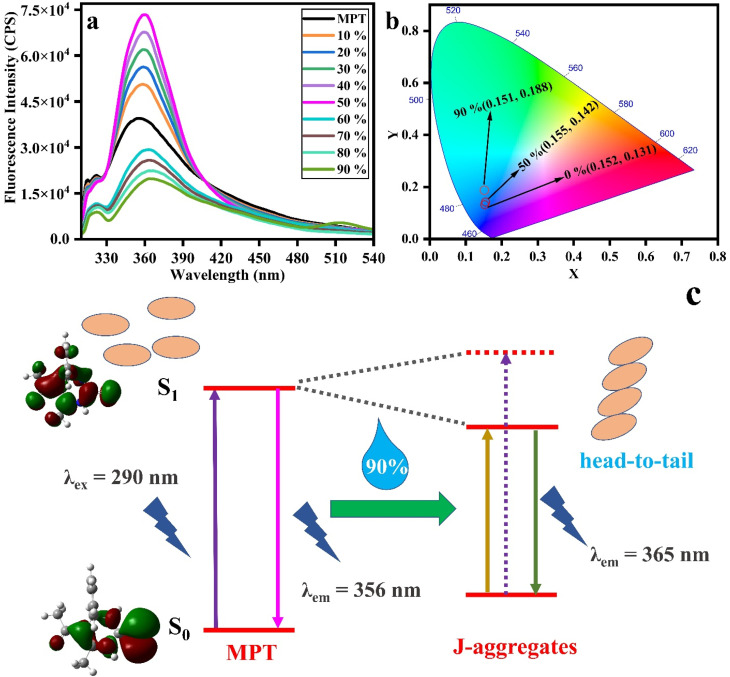
Emission spectra of MPT at 0% to 90% water fractions (a), CIE chromaticity diagram for different water fractions (b) and Kasha's rule explain that emission arises from the lowest excitonic state, validates J-aggregate formation (c) (excitation wavelength = 290 nm, concentration of sensor MPT = 30 µM).

The aggregation induced emission (AIE) behavior of sensor MPT was evaluated with increasing water fractions in binary solvent system. As shown in Fig. 1a, sensor MPT exhibited a weak emission around 356 nm in diluted DMF solutions when photoexcited at 290 nm. Upon gradually introducing water, a significant fluorescence enhancement accompanied by a slight red shift from 356 nm to 360 nm was observed as the water fraction increased from 0% to 50%, indicating the aggregation-induced emission (AIE) phenomenon. The increase in water content effectively restricts intramolecular rotations (RIR), which not only planarize the sensor MPT molecules, but also organizes its molecules and increases the overall dipole moment, which leads to the enhanced fluorescence intensity of sensor MPT. This AIE effect can be attributed to the formation of J-aggregates due to bathochromic shift in fluorescence intensity of sensor MPT, which was observed because of head to tail arrangement of molecules. In this process, water facilitates agglomeration, and its polar characteristics stabilize the resulting aggregates, leading to enhanced emission at slightly longer wavelengths.

As the water content increases from 60% to 90%, a significant decrease in the emission intensity of the sensor MPT was observed, accompanied by a slight redshift from 360 nm to 365 nm. At elevated water fractions, the sensor MPT molecules undergo complete planarization and commence π–π stacking, which effectively quenches their emission. The observed slight red shift can be attributed to an increase in the dipole moment of the sensor MPT molecules in its aggregated state. Furthermore, slight red shift indicates the formation of J-aggregates, resulting from the head-to-tail arrangement of sensor MPT molecules.^45,46^ Additionally, the relative emission intensity of sensor MPT was plotted against increasing water fractions, revealed a notable increase in emission intensity as the water fraction rose up to 50% (Fig. S3b). Based on these findings, a water fraction of 50% was selected for further photophysical studies.

Results obtained from the emission spectra were also drawn on CIE chromaticity diagram illustrating the impact of varying water content levels at 0%, 50% and 90%. These water contents are represented by red circles positioned within blue region at coordinates (0.152, 0.131), (0.155, 0.142) and (0.151, 0.188) represent a notable change in wavelength (Fig. 1b). As shown in Fig. 1c, according to Kasha's rule,^47^ fluorescence emission usually originates from the lowest excited singlet state (S_1_). In the given system, as the water fraction increases from 0% to 50%, a significant enhancement of the emission intensity is obtained with a slight red shift of the emission maximum from 356 to 360 nm. This enhancement can be attributed to the restriction of non-radiative decay processes *e.g.* intramolecular rotations which promote radiative decay from the S_1_ state. However, further increasing the water content from 60% to 90%, the emission intensity decreases, while the emission band shows a bathochromic shift towards 365 nm.

The reduction in fluorescence intensity at higher water fractions may result from aggregation-induced quenching, resulting from the formation of J-aggregates, which promote intermolecular π–π stacking and delocalization of excited states. Such aggregation not only introduces new non-radiative decay channels but also stabilizes the excited state, leading to a further red shift in emission. The presence of aggregates which enhances fluorescence intensity was further confirmed by dynamic light scattering analysis. The size of sensor MPT aggregates increases from 359 nm to 965 nm as the water content increases from 0% to 50% (Fig. S3c and d).

### 3.5 Optical sensing of H_2_O_2_

Prior to implementing sensor MPT in real-world applications, we assessed the UV-visible and fluorescence responses of the sensor (30 µM) to H_2_O_2_ in an aqueous medium (H_2_O : DMF, 1 : 1, v/v). We performed absorption studies with relevant target analytes such as H_2_O_2_, Na_2_SO_4_, NO_2_, K^+^, Mg^2+^, Fe^3+^, Cl^−^, OH^−^, ClO^−^, methanol (CH_3_OH), cysteine (Cys), proline (Pro), glutathione (GSH), glycine (Gly), and ascorbic acid (AA) to evaluate the selective sensing capabilities of the sensor MPT. The finding reveals minimal influence on the absorption band of the sensor MPT in the presence of these competing species (Fig. S4a), indicating that the sensor MPT demonstrates a strong potential for selective detection of H_2_O_2_. As depicted in Fig. S4b, the absorption peak experienced gradual enhancement upon the introduction of H_2_O_2_ at 322 nm (0–40 µM).

**Fig. 2:**
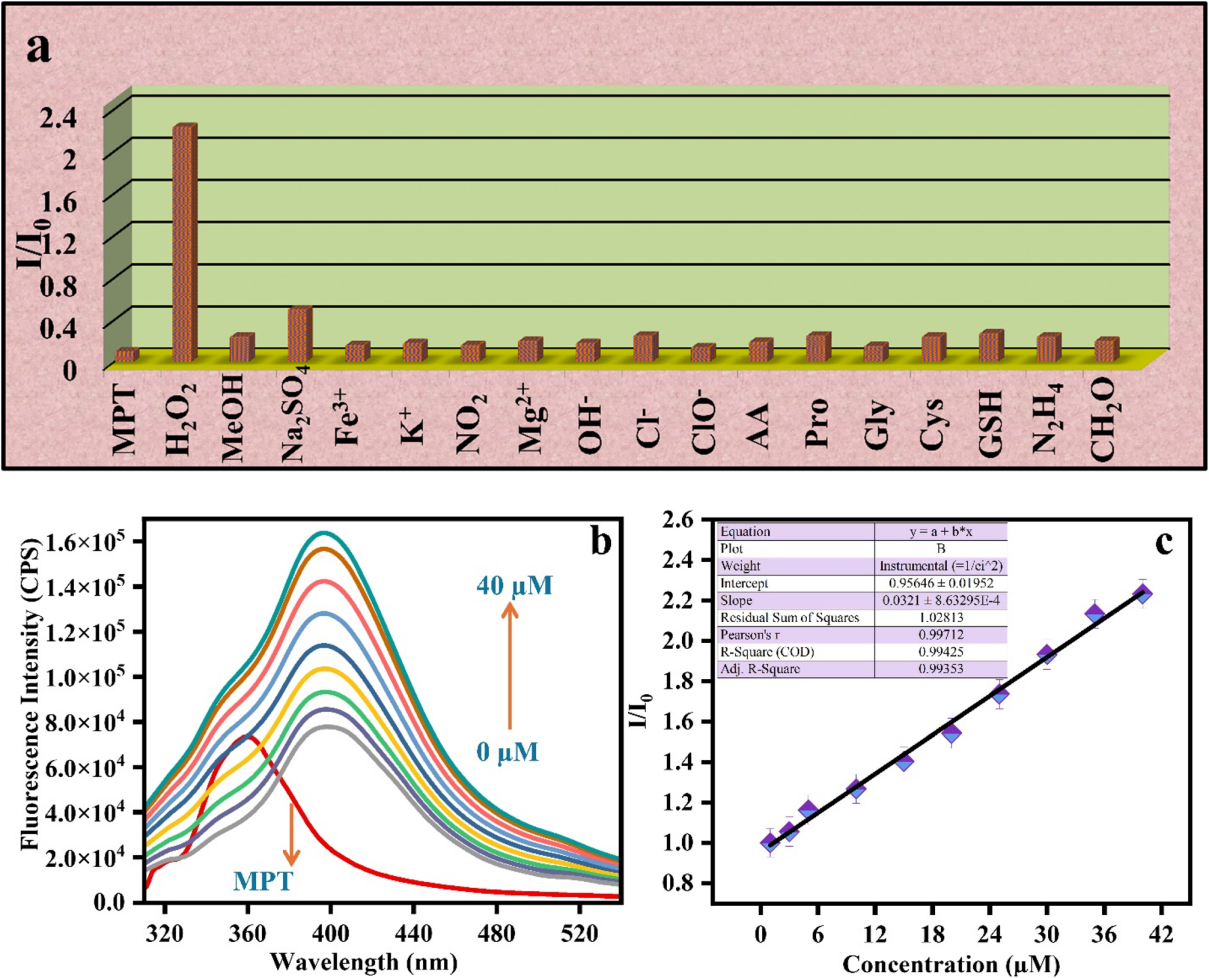
Relative emission of MPT in the presence of different analytes (40 µM) (a), sensor MPT against H_2_O_2_ (0–40 µM) and (b) 2D Benesi–Hildebrand plot of sensor MPT against H_2_O_2_ (c) (MPT = 30 µM in 1 : 1, v/v of DMF/H_2_O, *λ*_ex_ = 290 nm).

Further analysis was performed through fluorescence emission studies to explore the selective binding interaction of sensor MPT with H_2_O_2_. In this perspective, fluorescence spectra were recorded for various targeted analytes (40 µM) such as H_2_O_2_, Na_2_SO_4_, NO_2_, K^+^, Mg^2+^, Fe^3+^, Cl^−^, OH^−^, ClO^−^, HCHO (formalin, 37%), N_2_H_4_, methanol (CH_3_OH), cysteine (Cys), proline (Pro), glutathione (GSH), glycine (Gly), and ascorbic acid (AA) in an aqueous solution (H_2_O : DMF, 1 : 1, v/v) of sensor MPT (30 µM). Notably, the emission intensity of the sensor MPT exhibited a substantial increase upon the addition of H_2_O_2_, whereas only minor changes were observed with other competing analytes (Fig. S5). These results affirm the exceptional selectivity of the sensor MPT for H_2_O_2_, and relative emission intensity graph was also drawn against high concentrations of various targeted competing analytes (Fig. 2a).

Subsequent fluorescence titration experiments were conducted at different concentrations of H_2_O_2_ varying from 0 to 40 µM to assess the sensitivity of the sensor MPT towards H_2_O_2_. As illustrated in Fig. 2b, the emission of sensor MPT at 360 nm exhibited a remarkable enhancement alongside a notable bathochromic shift, moving from 360 nm to 400 nm upon incremental addition of H_2_O_2_ to sensor MPT. To quantitatively analyze the sensitivity, a 2D Benesi–Hildebrand plot was constructed correlating the relative fluorescence intensity of the MPT (*I*/*I*_0_) with the increasing concentration of H_2_O_2_ (0–40 µM). Within this concentration range, there was a significant linear relationship observed between *I*/*I*_0_ and H_2_O_2_ concentration, suggesting that a single mechanistic pathway is responsible for the sensing of H_2_O_2_ (Fig. 2c).

The binding association constant (*K*_a_) of the sensor MPT with H_2_O_2_ was determined from the linear portion of the Benesi–Hildebrand plot, yielding a value of 2.70 × 10^5^ M^−1^. This substantial *K*_a_ value indicates a strong affinity between the sensor MPT and H_2_O_2_. The linearity of the plot also facilitated the calculation of the limit of detection (LOD) and limit of quantification (LOQ) using the formula 3*σ*/*S* and 10*σ*/*S*, respectively, where *σ* represents the standard deviation and *S* corresponds to the slope of the calibration curve. As a result, the LOD and LOQ for MPT were determined to be 80.6 nM and 268 nM, respectively, demonstrating the sensitivity of the sensor. Furthermore, the sensing performance of sensor MPT is compared with previously documented sensors as illustrated in Table S1. This comparison clearly demonstrates the exceptional sensing capability of sensor MPT towards H_2_O_2_.

To further validate the sensor's sensitivity, we estimated the stoichiometric association through Job's plot analysis of the sensor MPT@H_2_O_2_ complex. In this experiment, the total concentration of the MPT was maintained at 30 µM while varying the mole fractions of H_2_O_2_ from 0 to 1.0 equivalents. The maximum relative emission occurred at the addition of 0.5 equivalents of H_2_O_2_, indicating a binding stoichiometry of 1 : 1 (Fig. S6). This finding suggests that one molecule of the MPT can effectively bind to one molecule of H_2_O_2_.

### 3.6 Mechanistic approach

The presence of hydrogen and oxygen atoms on H_2_O_2_ creates a promising pathway for effective electrostatic interaction with a suitable sensor MPT. Considering these factors, a remarkable fluorescent sensor MPT was developed with carbonyl and phenyl substitutions. The hydrogen of H_2_O_2_ is likely to develop strong hydrogen bonding interactions with acetyl carbonyl oxygen. At the same time, the oxygen atom of the analyte (H_2_O_2_) effectively coordinates with the hydrogen atom of freely rotatable phenyl ring of sensor MPT. Meanwhile, the oxygen atom in H_2_O_2_ form hydrogen bonding with the hydrogen atom of methyl in acetyl group. These exceptional hydrogen bonding interactions are responsible for extraordinary sensitivity of the sensor MPT towards H_2_O_2_, even in the presence of competing analytes. These strong interactions between sensor MPT and analyte H_2_O_2_ were also validated by a large bathochromic shift in emission wavelength from 360 nm to 400 nm. Moreover, hydrogen bonding is a key interaction in the sensing of hydrogen peroxide, as evidenced by theoretical studies including 3D isosurfaces, 2D reduced density gradient, and QTAIM analyses. These computational analyses further confirm the strong intermolecular interaction (hydrogen bonding) between sensor MPT and analyte H_2_O_2_. Hydrogen bonding between sensor MPT and hydrogen peroxide (H_2_O_2_) was clearly demonstrated through blue color patches in 3D iso-surface and blue spikes at −0.03 (product of second eigenvalue and electron density) in 2D-RDG. DLS titration experiments were also performed, which revealed a substantial shift in the hydrodynamic diameter of sensor MPT upon contact with H_2_O_2_. Particularly, the formation of the sensor MPT@H_2_O_2_ complex results in an enlargement in particle size from 965 nm to 1142 nm (Fig. S7).

**Fig. 3:**
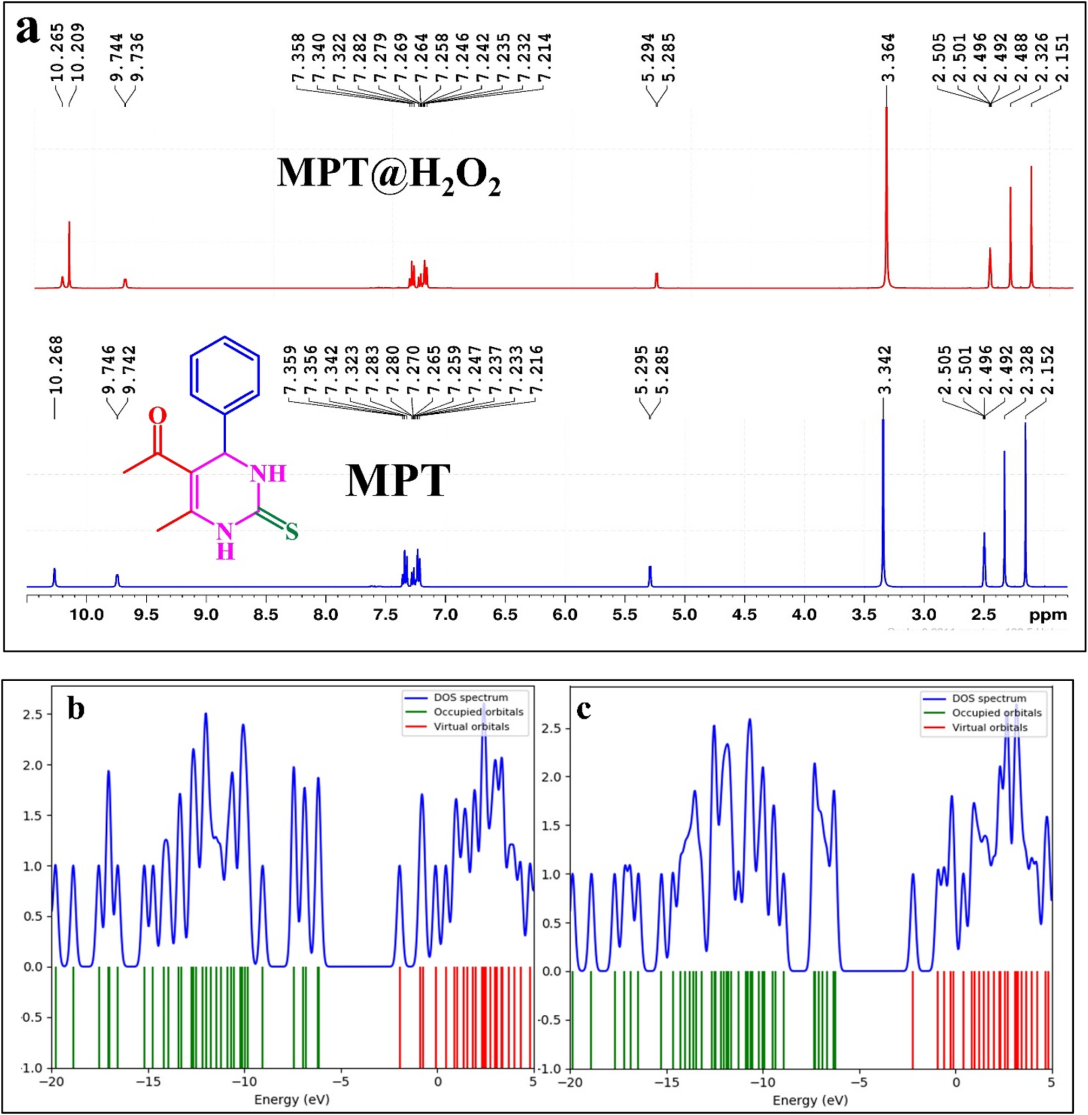
Stacked ^1^H-NMR titration spectra of sensor MPT and MPT@H_2_O_2_ in DMSO-*d*_6_ (a), density of state (DOS) spectra of both sensor MPT (b) and MPT@H_2_O_2_ complex (c) calculated at B3LYP/6-311G**.

Furthermore, a ^1^H NMR titration experiment was conducted in DMSO-*d*_6_ to investigate the nature of interaction whether covalent or non-covalent in nature between the sensor MPT and H_2_O_2_. To achieve this, ^1^H NMR spectra of the sensor MPT were recorded. Subsequently, 1.0 equivalent of H_2_O_2_ was added to the MPT sensor, and titration NMR spectra were collected. As shown in Fig. 3a, proton NMR titration experiment indicates that H_2_O_2_ does not undergo a chemical reaction with the sensor MPT as no significant variations in the chemical shifts of proton signals were observed. Furthermore, no new peaks or alterations in splitting patterns were observed. There is no evidence of covalent interaction between the sensor MPT and H_2_O_2_ molecules, suggesting that any potential interaction is of a non-covalent in nature.

The FTIR spectrum of the synthesized sensor MPT exhibits characteristics absorption bands that confirm the presence of functional groups in the sensor.^48,49^ The absorption band appearing in region 3290–3190 cm^−1^ are attributed to N–H stretching vibrations of the heterocyclic NH groups present in the sensor. The peaks appearing around 2980–2890 cm^−1^ correspond to aliphatic C–H stretching vibrations, while weak absorption near 3030 cm^−1^ indicates aromatic C–H stretching. A strong absorption band observed at 1680 cm^−1^ indicates the presence of C=O stretching vibration in the sensor framework. Bands in the 1600–1500 cm^−1^ region confirm aromatic C=C stretching vibrations and the absorption bands appearing in 1300–1100 cm^−1^ correspond to C–N stretching vibrations of the heterocyclic ring. A characteristic band in the lower frequency region (1150–1050 cm^−1^) is attributed to the C=S (thione) stretching vibration, supporting formation of the target sensor molecule. Upon addition of H_2_O_2_, noticeable spectral changes were observed. The N–H stretching band becomes broader and slightly shifted (3389–3225 cm^−1^), indicating hydrogen-bonding interaction^50^ between the sensor and H_2_O_2_. The carbonyl absorption band shifts from 1680 to 1633 cm^−1^, suggesting modification in the electronic environment of the carbonyl group during sensing interaction. Minor variations in the C–N and C=S regions further support interaction between the analyte and the active sites of the sensor molecule (Fig. S8).^51^

Mass spectrometric analysis provides additional confirmation of this interaction. The free sensor MPT exhibits a molecular ion peak at *m*/*z* 245.33, corresponding to the intact MPT molecule. Upon addition of H_2_O_2_, new peaks appear at *m*/*z* 277.08, corresponding to oxidation of MPT, indicating the formation of a stabilized interaction species in solution. The preservation of the parent fragmentation pattern alongside the emergence of higher *m*/*z* signals suggests complex formation rather than oxidative degradation of the sensor MPT, which is commonly observed in electrospray ionization mass spectrometry for hydrogen-bonded supramolecular assemblies.^52,53^ Based on these observations, H_2_O_2_ is proposed to interact with the sensor MPT*via* dual hydrogen bonding, in which H_2_O_2_ acts simultaneously as a hydrogen-bond donor and acceptor. The NH groups of MPT donate hydrogen bonds to the oxygen atoms of H_2_O_2_, while the sulfur atom of the thiocarbonyl group participates as a weak hydrogen bond acceptor. The formation of this hydrogen-bonded supramolecular complex alters the electronic environment of the sensor MPT ^54^ (Fig. S9).

The change in electron density over the sensor MPT molecule was clearly visible in FMO analysis. Moreover, it is theoretically supported by FMO, DOS, and NBO analyses performed using B3LYP as the functional and 6-311G** as the basis set. Before interacting with H_2_O_2_, the AIE-active sensor MPT likely undergoes restricted intramolecular rotation, as evidenced by its emission at 360 nm in an aggregated state. The electrostatic interaction (hydrogen bonding) between the sensor MPT and H_2_O_2_ likely restricts the intramolecular vibration and enhances the rigidity of the sensor MPT structure. This restriction and rigidity in the sensor MPT structure reduces non-radiative decay pathways and increase radiative decay, resulting in enhanced emission at 400 nm. Moreover, hydrogen bonding between sensor MPT and analyte (H_2_O_2_) stabilizes the electronic states of sensor, which results in a reduction in HOMO–LUMO energy gap that enhances the emission intensity of sensor MPT and results in a large bathochromic shift of 40 nm. This large Stokes shift in emission wavelength occurred due to a reduction in band gap, which was supported through FMO and DOS analyses of sensor MPT with and without H_2_O_2_.

### 3.7 Theoretical studies

**Fig. 4:**
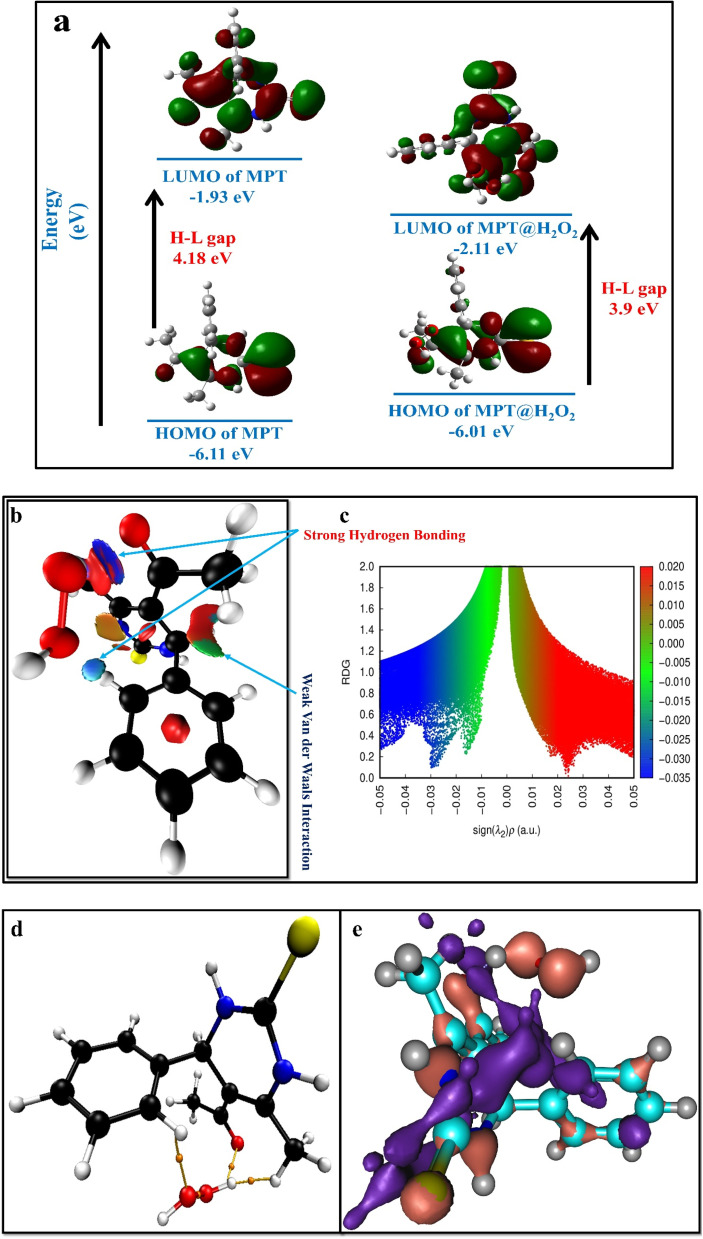
HOMO–LUMO energy gap of sensor MPT and MPT@H_2_O_2_ complex (a) calculated at functional B3LYP/6-311G**, 3D iso-surface for representation of intermolecular interactions in MPT@H_2_O_2_ complex generated at W-B97XD/6-311G** (b), and 2D reduced density gradient (RDG) of MPT@H_2_O_2_ complex (c), QTAIM analysis of MPT@H_2_O_2_ complex (d) and EDD analysis of MPT@H_2_O_2_ complex (e).

The optimization of structure of sensor MPT and MPT@H_2_O_2_ was carried out by using DFT/TD-DFT on functional B3LYP and 6-311G** basic set (Fig. S10). The energy of MPT@H_2_O_2_ was calculated using BSSE correction with the functional ωB97XD and the basis set 6-311G**. The 20.99 kcal mol^−1^ energy of MPT@H_2_O_2_ validates the suitability of site of interaction. The conductivity and resistivity of sensor MPT before and after its interaction with hydrogen peroxide were evaluated by utilizing FMO analyses. A clear change in electronic characteristics of sensor MPT was observed after its interaction with hydrogen peroxide (H_2_O_2_). The HOMO electronic density was located on pyrimidine ring, while LUMO on phenyl ring before the interaction of MPT with hydrogen peroxide (H_2_O_2_). After its interaction with hydrogen peroxide (H_2_O_2_) significantly variation in molecular orbitals was observed, the energy of LUMO orbital was reduced after interaction of MPT with hydrogen peroxide (H_2_O_2_), which also results in reduction of H–L energy gap after complexation of MPT@H_2_O_2_. H–L gap of sensor MPT was 4.11 eV, which was reduced to 3.39 eV after its complexation with hydrogen peroxide (Fig. 4a).

Furthermore, formation of new orbitals in density of state (DOS) spectra confirms the significant conductivity of MPT@H_2_O_2_, which verifies the sensitivity of sensor MPT for hydrogen peroxide (H_2_O_2_) (Fig. 3b and c).

To gain further insight into intermolecular interactions between the sensor MPT and hydrogen peroxide (H_2_O_2_), 2D reduced density gradient (RDG) and 3D isosurfaces were plotted to investigate the exact nature/types of electrostatic interactions. In 3D isosurfaces blue color demonstrates the presence of hydrogen bonding between hydrogen peroxide and sensor MPT molecule. A clear hydrogen bonding between hydrogen of hydrogen peroxide (H_2_O_2_) and acyl carbonyl oxygen on pyrimidine ring of sensor MPT molecule, while oxygen of hydrogen peroxide (H_2_O_2_) shows hydrogen bonding with hydrogen of phenyl ring and increases the rigidity of the molecular structure of sensor MPT. Along with blue patches, green and red patches are also observed, with green patches representing weak intermolecular forces and red patches showing repulsive forces within the aromatic rings (Fig. 4b).

A 2D intermolecular forces graph was plotted between sign(*λ*_2_)*ρ*(a.u.) on *x*-axis and reduced density gradient (RDG) on *y*-axis respectively. Blue spikes indicate hydrogen bonding between sensor MPT and hydrogen peroxide (H_2_O_2_). Weak to strong intermolecular hydrogen bonding was identified by the existence of blue spikes in the region of −0.015 to −0.033 a.u. (Fig. 4c).

QTAIM (Fig. 4d) was first stated by Bader *et al.* and used to evaluate nature of bonds.^55^ Various angles of BCPs (bond critical points) were investigated to recognize nature of intermolecular interactions. QTAIM demonstrates the exact nature of intermolecular electrostatic interactions between atoms of sensor MPT and analyte (H_2_O_2_). Table S2 shows the different parameters of QTAIM that demonstrate the interaction between MPT, and hydrogen peroxide is strong hydrogen bonding. Natural bonding orbitals (NBO) analyses were performed to evaluate charge transfer; quantity of charge transfer between sensor MPT and hydrogen peroxide was quantified as −0.0231*e*. EDD analyses were carried out to obtain a visual demonstration between sensor MPT and hydrogen peroxide (H_2_O_2_) (Fig. 4e).

### 3.8 Interference studies

To assess the selectivity of the fluorescent sensor MPT for H_2_O_2_, we conducted sensing studies in the presence of various potential interfering ions. The fluorescence response of MPT was found to be unaffected by these interfering species, demonstrating its specificity for H_2_O_2_ (Fig. S11a and b). We further evaluated the sensing capability of MPT across a pH range of 4–10 to determine its suitability for on-site applications. Notably, despite the variations in pH, the enhancement response of MPT upon H_2_O_2_ interaction remained stable (Fig. S12a). Additionally, we investigated the impact of temperature on the sensing performance by varying the temperature from 20 °C to 80 °C within DMF:H_2_O matrix. We limited the temperature increase to avoid water boiling and the results indicated that the sensing response of MPT remained consistent across the tested temperature range (Fig. S12b).

The enhancement response of the MPT sensor was also evaluated over a time range of 10–90 seconds, revealing that its enhancement efficiency for H_2_O_2_ remained stable, indicating a rapid response characteristic of the MPT sensor (Fig. S13a). Additionally, a photostability assessment was performed where MPT sensor was subjected to high energy radiation, demonstrating considerable stability with no observable deterioration in its H_2_O_2_ sensing capability (Fig. S13b). The MPT sensor exhibited a robust fluorescence response that was unaffected by variations in pH, fluctuation in temperature and photobleaching effects, affirming its suitability for practical applications due to its rapid response time.

## 4. Applications

### 4.1 H_2_O_2_ responsive test kits

In the realm of organic light emitting diodes (OLEDs) and fluorescent sensors, there has been substantial focus on the advancement of solid-supported display materials. The significant emission of a fluorophore in a solid state is crucial for device fabrication intended for use in OLEDs.^56^

**Fig. 5:**
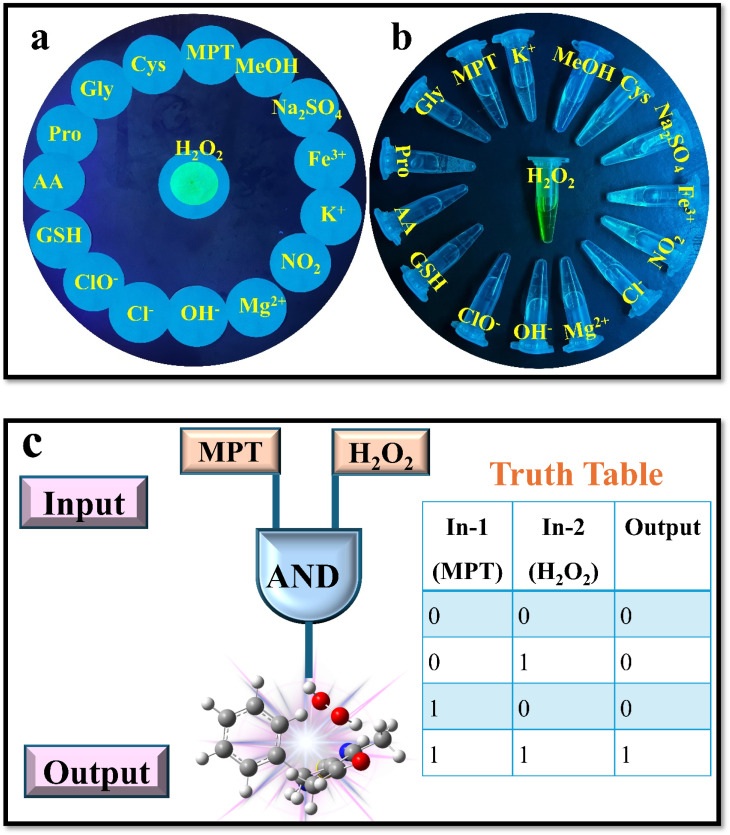
Colorimetric analysis of MPT against H_2_O_2_ detection in solid and liquid phase (a and b) and construction of logic gate along with its truth table for real time monitoring of analyte (c).

Considering this, the favorable fluorescence enhancement response of MPT to H_2_O_2_ has motivated the creation of effective solid-supported emitters for the simplified detection of H_2_O_2_. In this context, H_2_O_2_-responsive test kits were systematically developed using the dip coating method with Whatman filter paper strips that showed blue emission when exposed to UV radiation (365 nm). However, the application of a single drop of H_2_O_2_ solution onto the sensor's coated paper strips enhanced the emission of MPT (Fig. 5a). Meanwhile the introduction of other targeted analytes did not affect the emission of MPT under UV radiation. Thus, it is clearly demonstrated that H_2_O_2_-responsive test strips of MPT could potentially serve for easy, reliable and cost-effective detection of H_2_O_2_ in real samples.

### 4.2 Visual sensing of H_2_O_2_ in solution phase

The visual detection abilities of sensor MPT for H_2_O_2_ were evaluated to determine their practical relevance and selectivity. In this context, 50 µM of each targeted analytes including H_2_O_2_, Na_2_SO_4_, NO_2_, K^+^, Mg^2+^, Fe^3+^, Cl^−^, OH^−^, ClO^−^, methanol (CH_3_OH), cysteine (Cys), proline (Pro), glutathione (GSH), glycine (Gly), and ascorbic acid (AA) were added into a 30 µM solution of MPT in a 1 : 1, v/v of DMF/H_2_O solvent mixture (Fig. 5b). Under UV 365 nm, a significant color change was noticed, the emission color of sensor MPT enhanced in the presence of H_2_O_2_. Conversely, the addition of other targeted analytes did not cause any change in the color of the sensor MPT when subjected to UV radiation, confirming the sensor's exceptional selectivity and practical sensing capability for detecting H_2_O_2_. Considering all of this, the rapid colorimetric detection ability of MPT was effectively utilized for on-site detection of H_2_O_2_.

### 4.3 Vapor phase detection of H_2_O_2_

H_2_O_2_ plays a role in the creation of commonly used peroxide-based explosives and can also emerge as a byproduct from the decomposed of these explosives. Thus, detection H_2_O_2_ vapors may enhance security surveillance. Sensor MPT coated strips were developed for the detection of H_2_O_2_ vapors, as depicted in Fig. S14. The pristine sensor MPT exhibited a light cyan and blue under 254 nm and 356 nm UV light. However, after being exposed to H_2_O_2_, the sensor emission changed to yellow and sparking light yellow. Consequently, the alteration in the sensor emission was noticeable to the naked eye, making it suitable for on-site detection of H_2_O_2_ vapors.

### 4.4 Construction of logic gates

Significant advancements have been achieved in advanced computing systems to interpret or combine fluorescence spectral data into chemically coded outputs through the use of logic gates. Various innovative electronic devices based on semiconductor logic gates have been developed for the real-time monitoring of specific analytes.^57^ A logic gate was built using AND gates with MPT alone and MPT@H_2_O_2_ complex as input 1 (In-1) and input 2 (In-2) and fluorescence emission at 400 nm as the output. Emission wavelength equal to threshold limit is indicated as “1” representing the ON state. Conversely an emission wavelength below the threshold value is labeled as “0” which corresponds to the OFF state. The logic device displayed an ON state (OUT, 1) only when MPT@H_2_O_2_ (In-2) was in the ON position. A truth table was created based on the fluorescence response of MPT in the presence and absence of H_2_O_2_, illustrating various input combinations and their corresponding output responses (Fig. 5c). Considering all these findings, it is expected that the functions of MPT and MPT@H_2_O_2_ in the development of the logic gate may be significant in the area of nano-devices and molecular electronics.

### 4.5 Sensing of hydrogen peroxide (H_2_O_2_) in real samples

Excessive use of hydrogen peroxide in various commercial products often contaminates commonly used items, including food and water. Therefore, the practical applications of MPT were expanded to enable the qualitative and quantitative determination of hydrogen peroxide in commercial products, including water, milk, and juice samples. Industrial samples were collected from the heavy-industrial zone in Lahore, Pakistan, and processed to remove unwanted solid particles. Different concentrations of hydrogen peroxide were spiked into real commercial samples. Emission spectra of MPT were recorded with and without the addition of various concentrations of hydrogen peroxide to spiked samples. The results were compared with the emission enhancement results of MPT obtained in the presence of laboratory hydrogen peroxide samples, which provided the recovery quantification (Table S3). Recovery outcomes showed no significant variation across the spiked concentrations of hydrogen peroxide, and recoveries ranged from 95% to 97.5% with RSDs ≤ 2.13%. These outcomes indicate that our MPT has great potential for determining hydrogen peroxide in real commercial samples with high precision.

The concentration of hydrogen peroxide in commercially available products, including hair bleach, mouthwash, and disinfectant, was measured, which were purchased from a local drug store. The hair dye was claimed to contain 6% hydrogen peroxide, while the mouthwash and disinfectant contained 1.5% and 3% hydrogen peroxide, respectively. After preparing stock solutions of all products, various concentrations of hydrogen peroxide (0–4%, v/v) were titrated against MPT. All recovery outcomes are shown in Table S4. Concentration of hydrogen peroxide determined in disinfectant, mouthwash, and hair dye were 0.86 M (2.98%), 0.44 M (1.50%), and 1.75 M (5.99%), respectively. The response time of MPT to hydrogen peroxide, even in commercial real samples, was observed to be 25 s, which is extremely quick for sensing hydrogen peroxide concentration.

## 5. Conclusion

In this research, a fluorescent sensor based on pyrimidine, named MPT was utilized for detection of dangerous H_2_O_2_ with very low detection limit of 80.6 nM. The sensor MPT was carefully modified with twisted group that contributed to aggregation-induced emission characteristics. The interaction of H_2_O_2_*via* hydrogen bonding with the AIE sensor MPT inhibits intramolecular rotations and increases the rigidity of structure, which results in bathochromic shift of 40 nm with significant enhancement in the sensor's emissions at 400 nm. The importance of our sensor MPT is highlighted by its high selectivity, distinct sensing mechanism (hydrogen bonding) and the noticeable emission enhancement following its interaction with H_2_O_2_. Comprehensive TD-DFT studies corroborate this sensing method. It is anticipated that intramolecular rotational restrictions as a sensing technique could inspire the creation of new sensors for the selective detection of various analytes. Additionally, the sensor MPT showed an excellent sensing response to H_2_O_2_ vapors, design of logic circuit. Sensor MPT was also able to determine H_2_O_2_ in real samples which enhances its potential for on-site H_2_O_2_ detection. We are confident that this research will provide a straightforward strategy for designing highly effective fluorescent sensors for the selective detection of H_2_O_2_.

## Conflicts of interest

The authors declare that they have no known competing financial interests or personal relationships that could have appeared to influence the work reported in this paper.

## Supplementary Material

RA-OLF-D6RA02128J-s001

## Data Availability

The supporting data have been provided as a part of the supplementary information (SI). Supplementary information: NMR, mass spectrometry data, emission and absorption spectra of sensor MPT, optimized structures of sensor MPT and MPT@H_2_O_2_ complex. See DOI: https://doi.org/10.1039/d6ra02128j.
