# Supplementary material for: Construction of AIE active fluorescent sensor for highly specific sensing of hydrogen peroxide through turn on fluorescence emission
Source: RSC Adv. 2026 Jul 2;16(34):32618–31. doi: 10.1039/d6ra02128j (PMC13325429; doi:10.1039/d6ra02128j)

Supplementary Information (SI) for RSC Advances.
This journal is © The Royal Society of Chemistry 2026

Supporting Information

Construction of AIE Active Fluorescent Sensor for Highly Specific Sensing of Hydrogen Peroxide Through Turn On Fluorescence Emission

Iqra Mustafa[a,1], Muqadas[a,1], Alam Shabbir[a], Mohammed A. Assiri[b,c], Umar Farooq[a,] *, Sohail Anjum Shahzad[a,] *

[a] *Department of Chemistry, COMSATS University Islamabad, Abbottabad Campus, University Road, Abbottabad 22060, Pakistan*

[b] *Department of Chemistry, Faculty of Science, King Khalid University, P.O. Box 9004, Abha 61413, Saudi Arabia*

[c] *Central Labs, King Khalid University, AlQura'a, Abha, P.O. Box 960, 61413, Saudi Arabia*

* **Corresponding authors:** Department of Chemistry, COMSATS University Islamabad, Abbottabad Campus, University Road, Abbottabad 22060, Pakistan

E-mail addresses: umarf@cuiatd.edu.pk (U. Farooq); sashahzad@cuiatd.edu.pk (S. A. Shahzad)

[1]These authors contributed equally to this work.

Table of Contents

SI-1. Instruments and reagents…....4

Figure S1. ESI-MS spectrum of synthesized sensor **MPT**…4

Figure S2. Emission spectra of sensor **MPT** at different concentrations (5–50 μM) (a), absorption spectra of sensor **MPT** at different concentrations (5–50 μM) (b) and absorption and emission spectra of **MPT** in DMF (c). (Excitation wavelength= 290 nm)…...5

Figure S3: Emission spectra of sensor **MPT** for variety of solvent environment (a), relative emission intensity of the **MPT** in increasing water fractions (0 – 90 %) (b), DLS analysis at 0 % and 90 % water fraction (c and d) (Excitation wavelength = 290 nm, concentration of sensor **MPT** = 30 μM)…6

Figure S4: Change in absorption spectra of sensor **MPT** in the presence of target competing analytes (40 μM) (a) and absorption response of **MPT** against increasing concentration of H_2O_2 (b). (Excitation wavelength= 290 nm, Concentration of **MPT** 30 μM in 1:1, v/v of DMF/H_2O).…6

Figure S5: Emission behavior of sensor **MPT** with the addition of H_2O_2 and their competing analytes (40 μM). (Excitation wavelength = 290 nm, Concentration of **MPT** 30 μM in 1:1, v/v of DMF/H_2O)…...7

Figure S6: Job's plot of sensor **MPT@**H_2O_2 complex to evaluate the stoichiometric association (λ_{ex}=290 nm). …7

Figure S7: DLS analysis of sensor **MPT** (a) and with H_2O_2 (b) to confirm the interaction between two…8

Figure S8: FTIR spectrum of sensor **MPT** (a) and **MPT@**H_2O_2 complex (b)…8

Figure S9: LC-MS titration spectra of sensor **MPT** (a) and **MPT@**H_2O_2 complex (b)…9

Figure S10: Optimized structures of sensor **MPT** and **MPT@**H_2O_2 complex with DFT/TD-DFT on functional B3LYP and basic set 6-311G**…9

Figure S11: Emission spectra of sensor **MPT** and **MPT@**H_2O_2in the presence of cations and anions(a and b)…..10

Figure S12: Effect of pH (a) and temperature (b) on enhancement response of sensor **MPT** towards H_2O_2…11

Figure S13: Relative fluorescence enhancement of **MPT** for H_2O_2 in the time interval of 10-90 sec (a) and photostability test of sensor **MPT** towards H_2O_2 (b). …11

Figure S14: Response of fluorescent paper strip of sensor **MPT** before and after exposure to H_2O_2 vapors. …12

Table S1. Comparison of **MPT** sensor with already reported H_2O_2 sensor.................................13

Table S2. Representation of BCPs of **MPT**@H_2O_2...13

Table S3. Results for the quantification of hydrogen peroxide in real samples……………….14

Table S4. Results for the determination of hydrogen peroxide (H_2O_2) in commercially available real samples……………………………………………………………………………14

SI-1. **Instruments and reagents**

The sensor was characterized by performing ^{1}H-NMR at 400 MHz, ^{13}C-NMR and DEPT-135 at 100 MHz with the help of Bruker Avance III NMR spectrometer employing DMSO-d_6 as solvent. NMR titration experiments were carried out in DMSO-d_6. The spectrofluorometer (FluoroMax-Plus-P-C, Horiba Jobin Yvon Technology, USA) and spectrophotometer (Shimadzu UV-1601) were used to record the fluorescence emission and absorption spectra, respectively. The fluorescence studies were performed on solutions of probe **MPT** in HPLC grade DMF to detect the anticipated analyte. All chemicals and reagents, purchased from Daejung Chemicals & Metals (Korea), Oakwood Chemicals (USA), Sigma Aldrich (USA), and Alfa Aesar (UK), were employed directly without any further purification. The reagents and chemicals include H_2O_2, Na_2SO_4, NO_2, K^+, Mg^{2+}, Fe^{3+}, Cl^-, OH^-, ClO^-, methanol (CH_3OH), formalin (37%), cysteine (Cys), proline (Pro), glutathione (GSH), glycine (Gly), ascorbic acid (AA), *n*-hexane, ethanol, acetonitrile (CH_3CN), dichloromethane (DCM) and *N,N*-dimethylformamide (DMF). All spectral measurements were conducted at ambient temperature (25 ± 1°C) in DMF using a cuvette with a path length of 1cm.

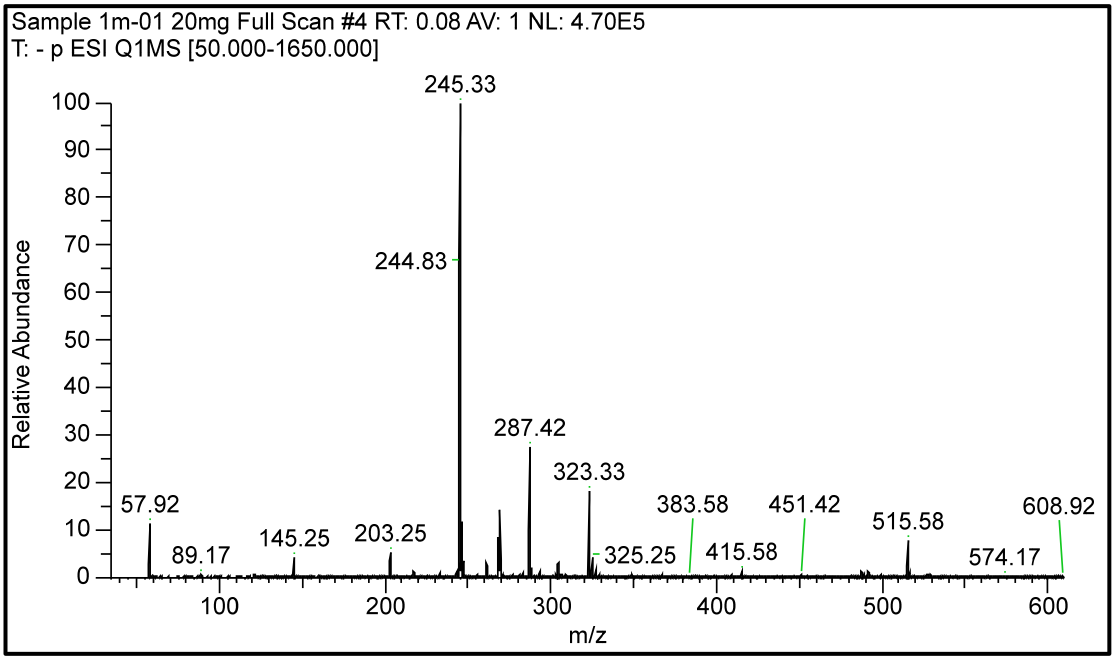

Figure S1: ESI-MS spectrum of synthesized sensor **MPT**.

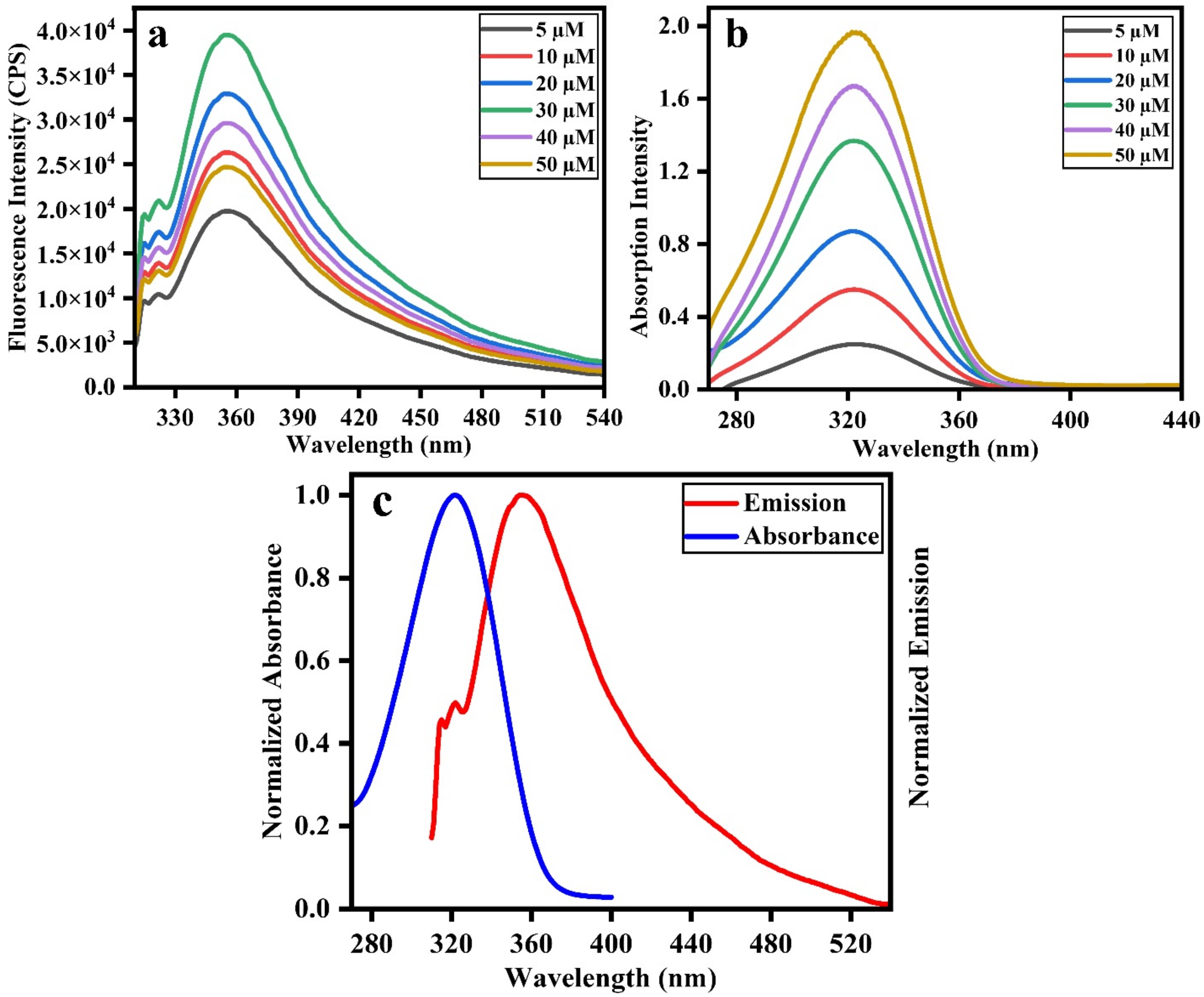

Figure S2: Emission spectra of sensor **MPT** at different concentrations (5–50 µM) (a), absorption spectra of sensor **MPT** at different concentrations (5–50 µM) (b) and absorption and emission spectra of **MPT** in DMF (c). (Excitation wavelength = 290 nm)

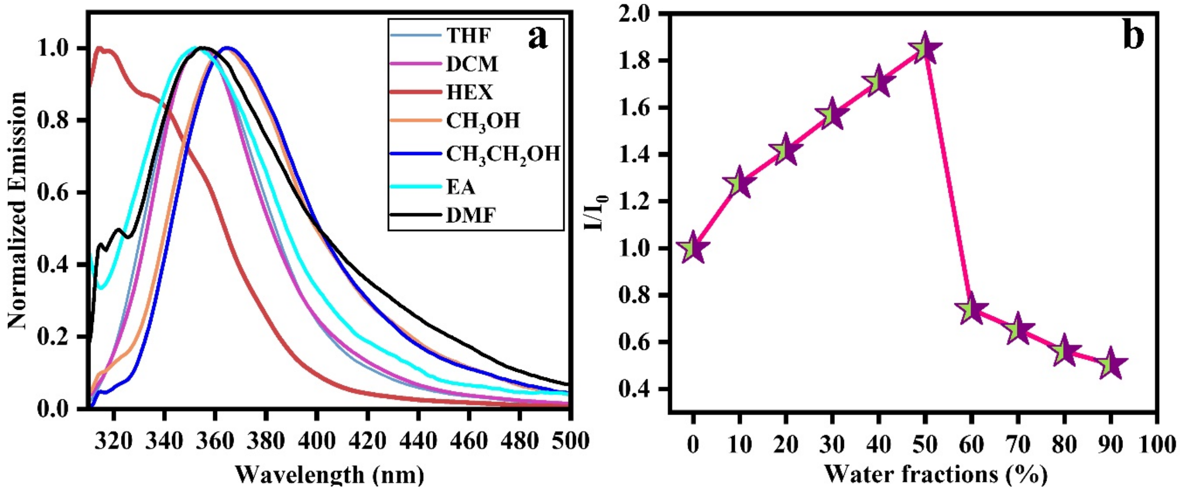

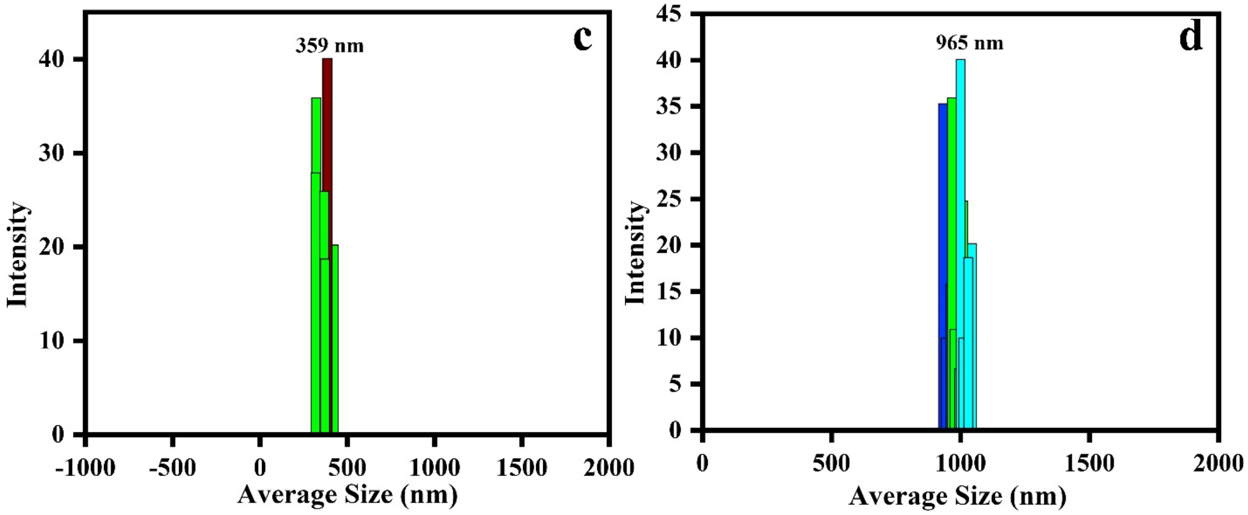

Figure S3: Emission spectra of sensor **MPT** for variety of solvent environment (a), relative emission intensity of the **MPT** in increasing water fractions (0 – 90 %) (b), DLS analysis at 0 % and 50 % water fraction (c and d) (Excitation wavelength = 290 nm, concentration of sensor **MPT** = 30 μM).

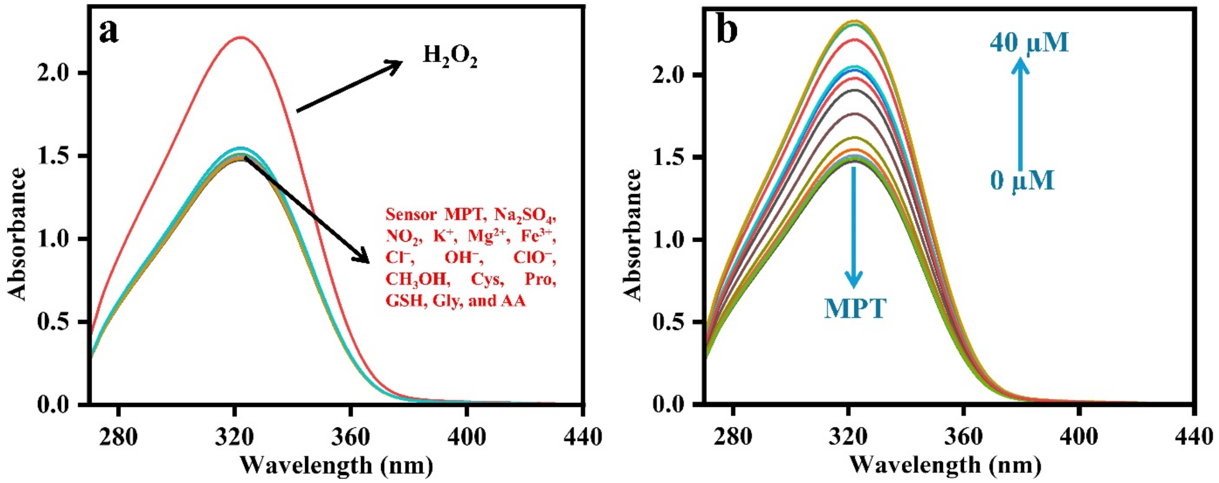

Figure S4: Change in absorption spectra of sensor **MPT** in the presence of target competing analytes (40 μM) (a) and absorption response of **MPT** against increasing concentration of H_2O_2 (b). (Excitation wavelength= 290 nm, Concentration of **MPT** 30 μM in 1:1, v/v of DMF/H_2O).

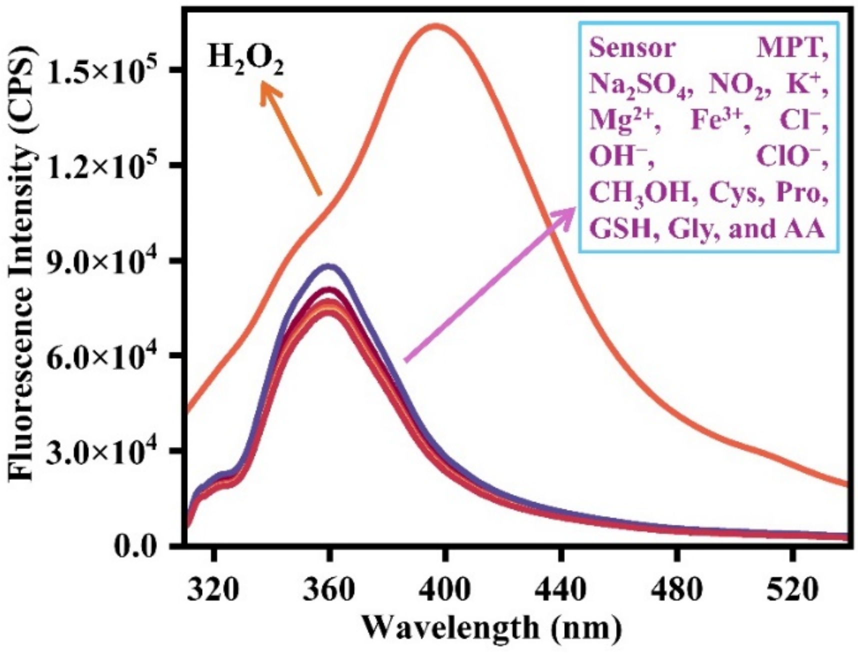

Figure S5: Emission behavior of sensor **MPT** with the addition of H_2O_2 and their competing analytes (40 µM). (Excitation wavelength = 290 nm, Concentration of **MPT** 30 µM in 1:1, v/v of DMF/H_2O).

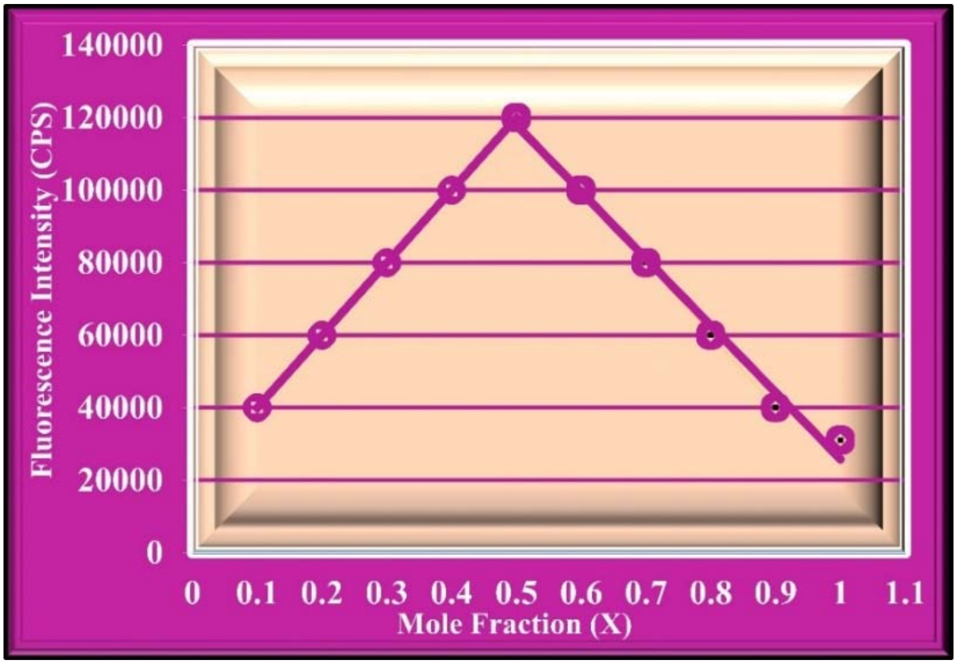

Figure S6: Job's plot of sensor **MPT**@H_2O_2 complex to evaluate the stoichiometric association (λ_{ex} = 290 nm).

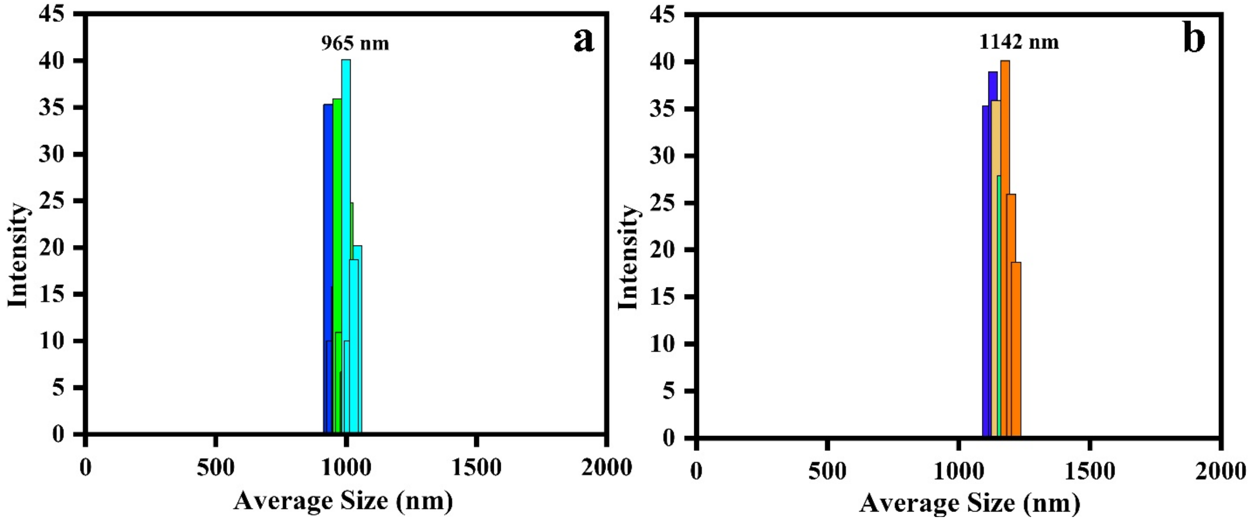

Figure S7: DLS analysis of sensor **MPT** (a) and with H_2O_2 (b) to confirm the interaction between two.

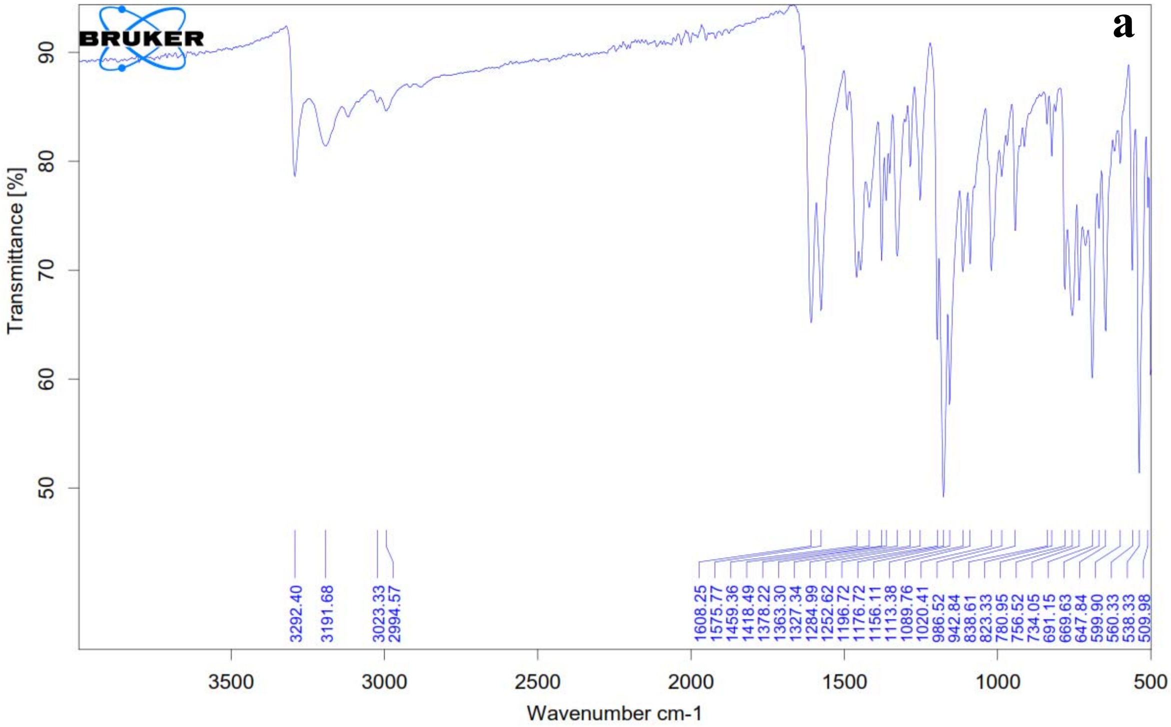

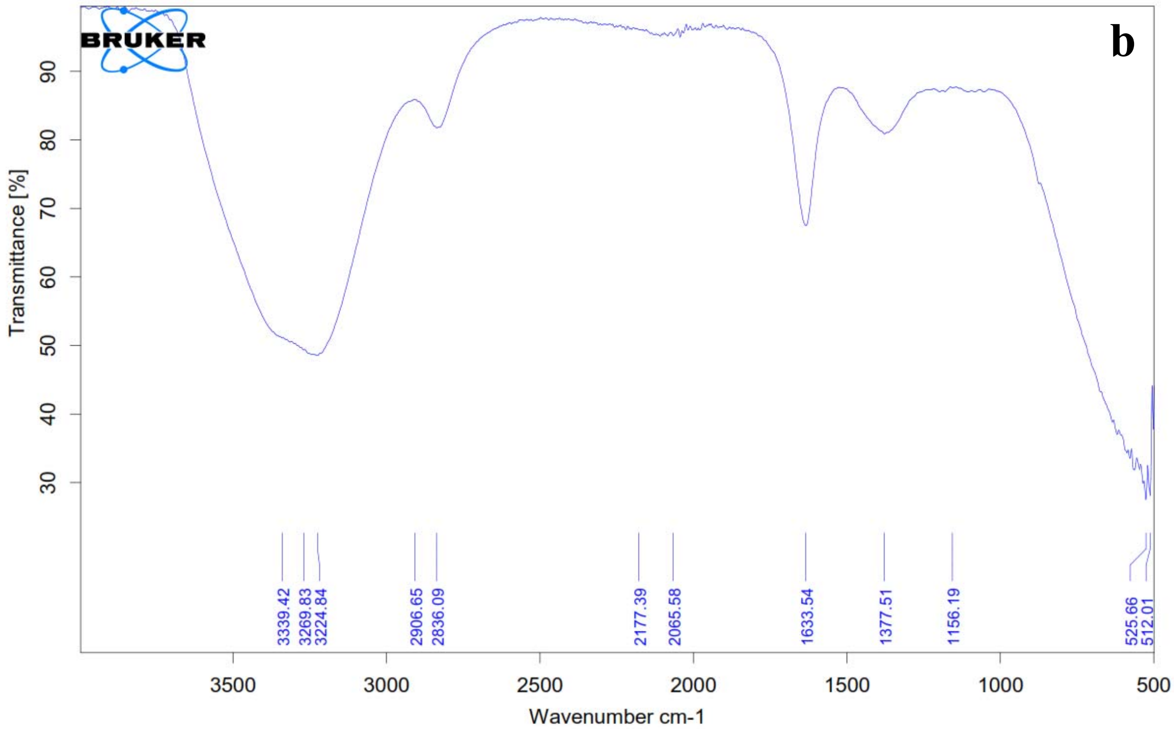

Figure S8: FTIR spectrum of sensor **MPT** (a) and **MPT**@H_2O_2 complex (b).

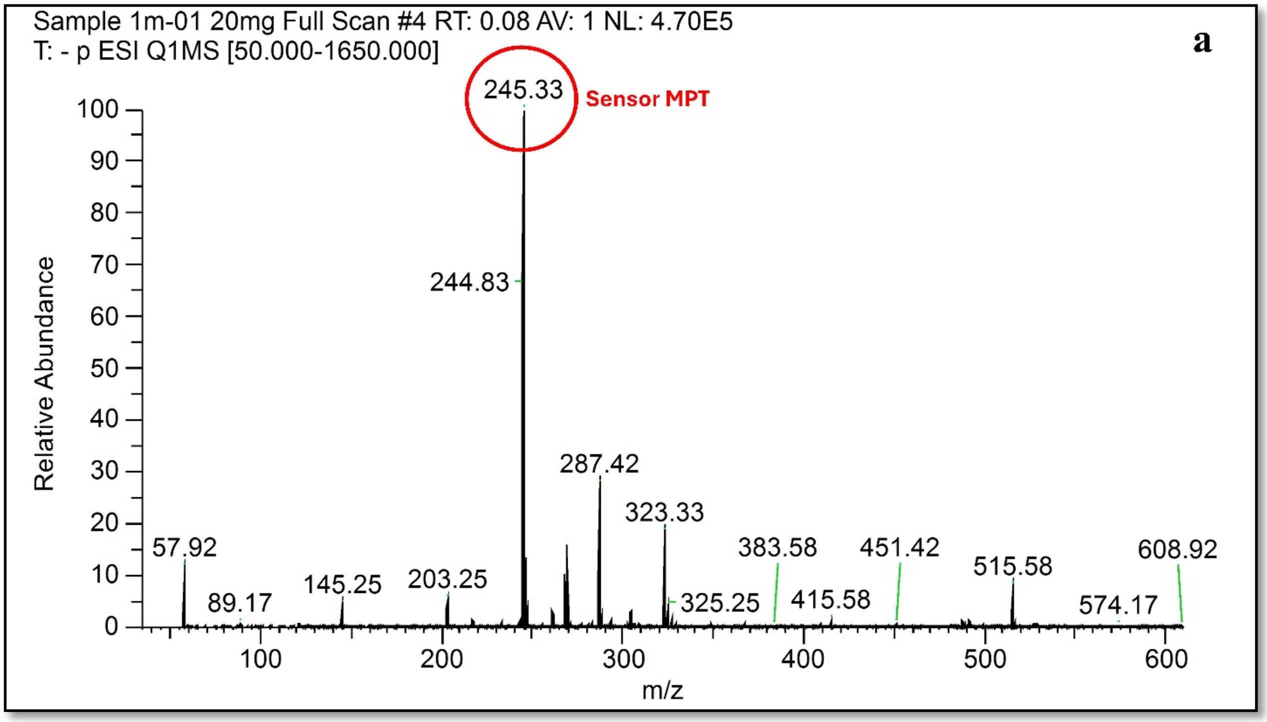

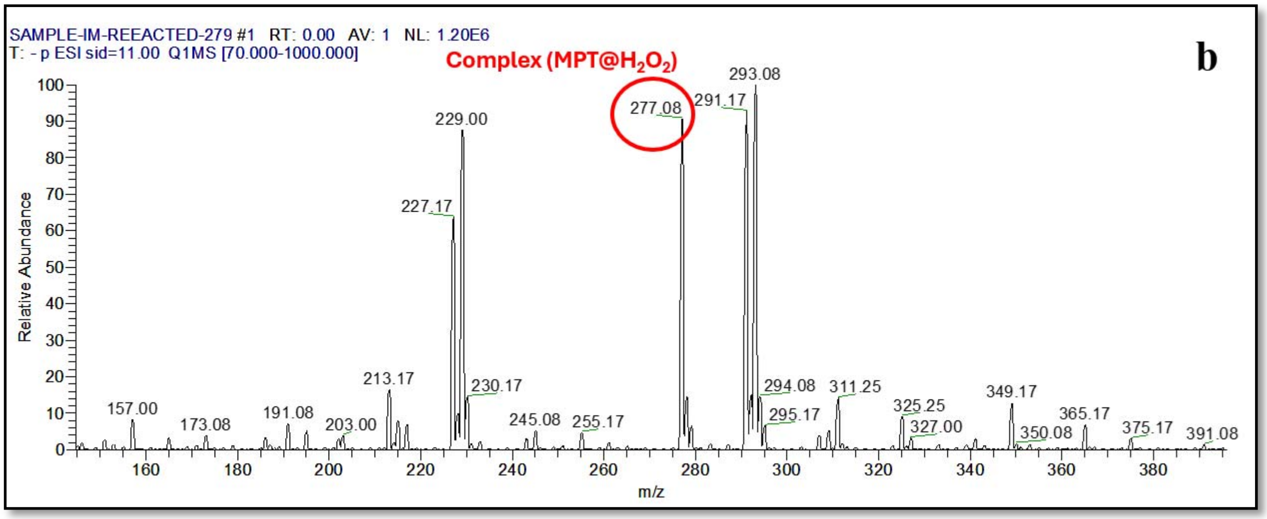

Figure S9: LC-MS titration spectra of sensor **MPT** (a) and **MPT**@H_2O_2 complex (b).

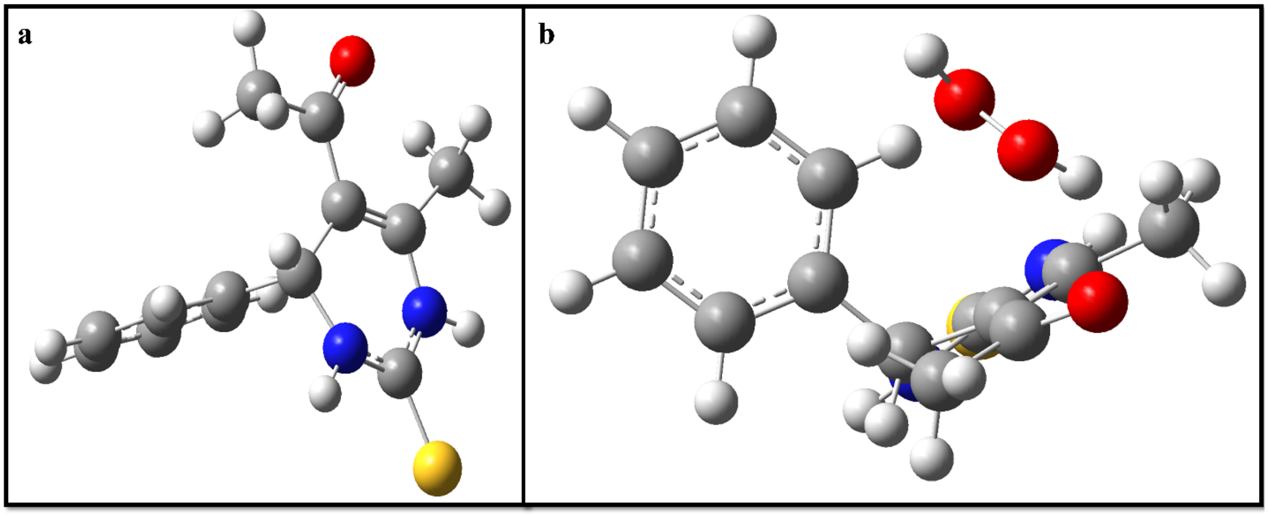

Figure S10: Optimized structures of sensor **MPT** and **MPT**@H_2O_2 complex with DFT/TD-DFT on functional B3LYP with basis set 6-311G**.

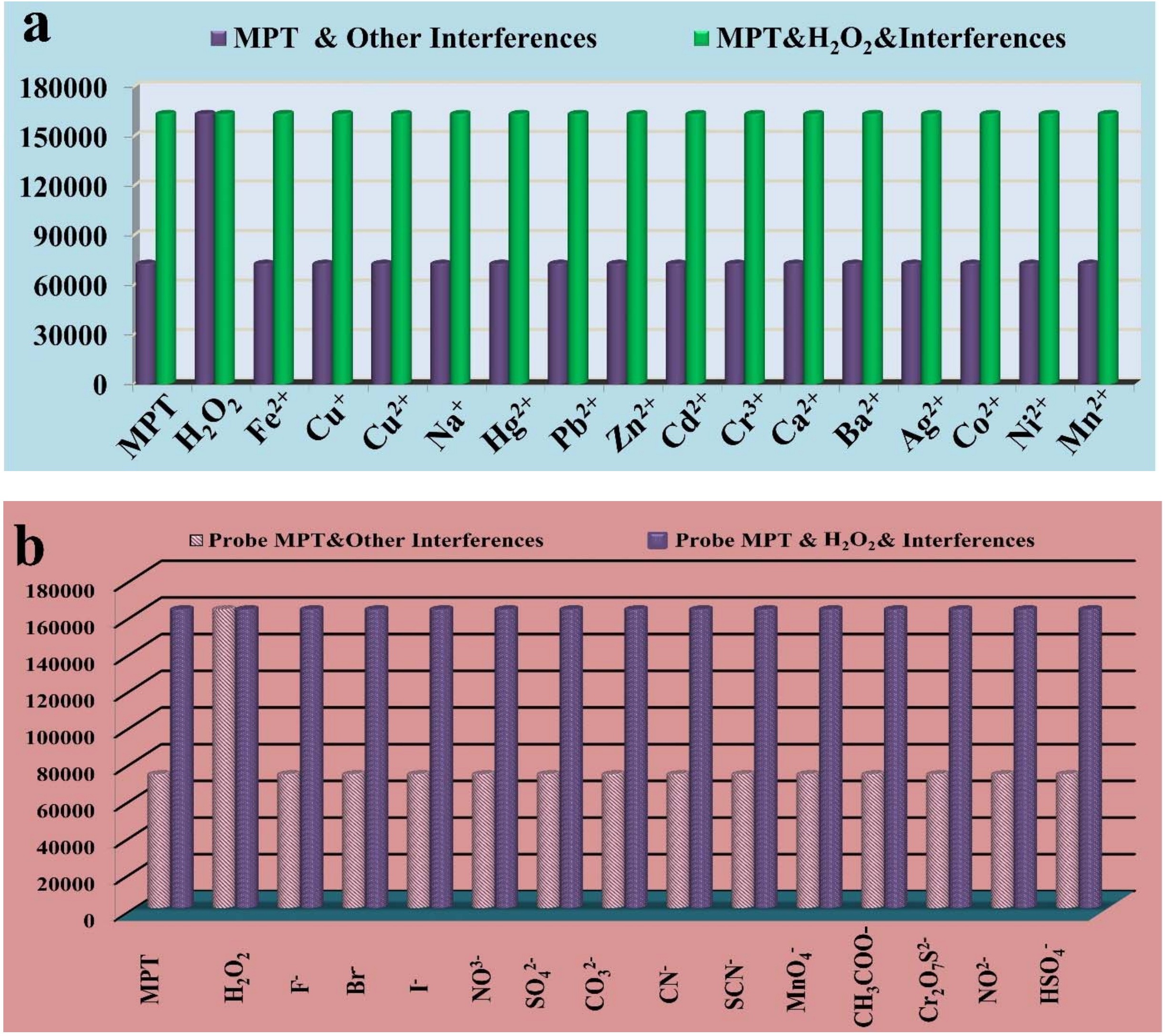

Figure S11: Emission spectra of sensor **MPT** and **MPT**@H2O2in the presence of cations and anions (a and b).

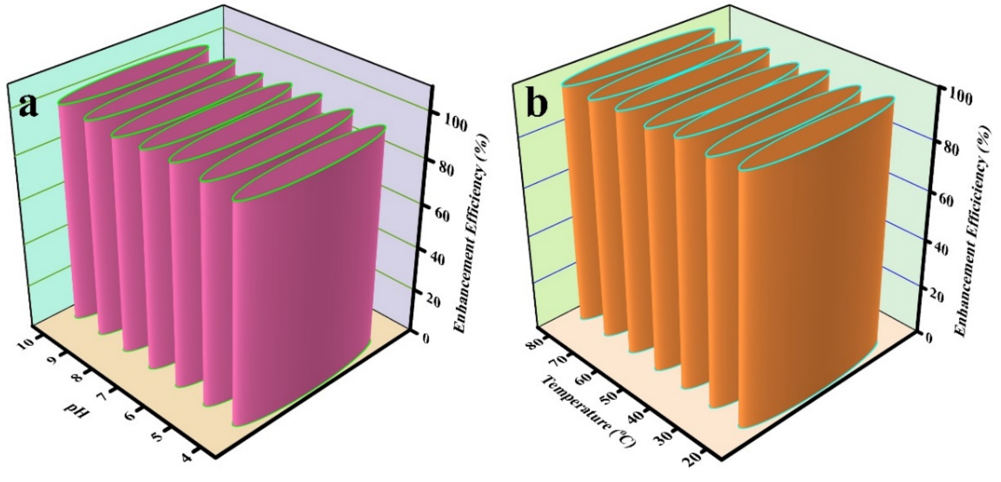

Figure S12: Effect of pH (a) and temperature (b) on enhancement response of sensor **MPT** towards H_2O_2.

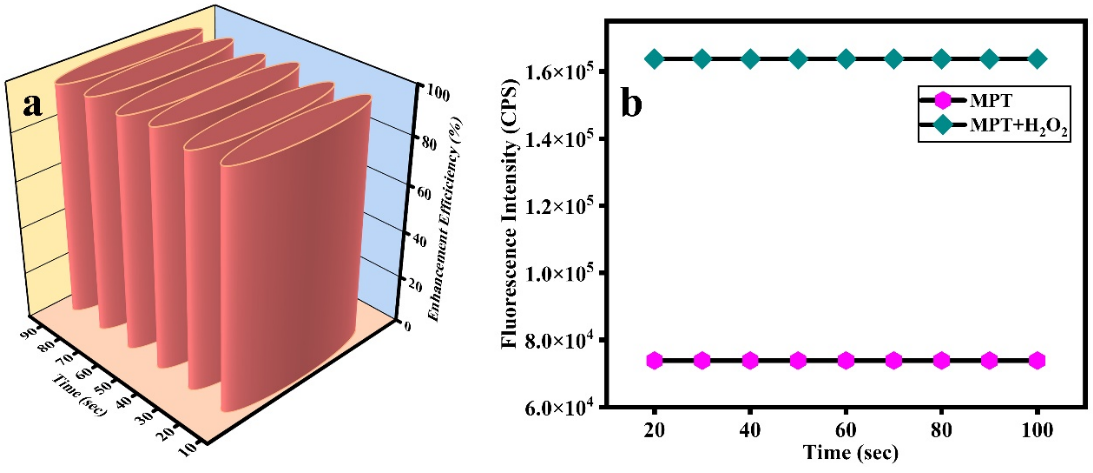

Figure S13: Relative fluorescence enhancement of **MPT** for H_2O_2 in the time interval of 10-90 sec (a) and photostability test of sensor **MPT** towards H_2O_2 (b).

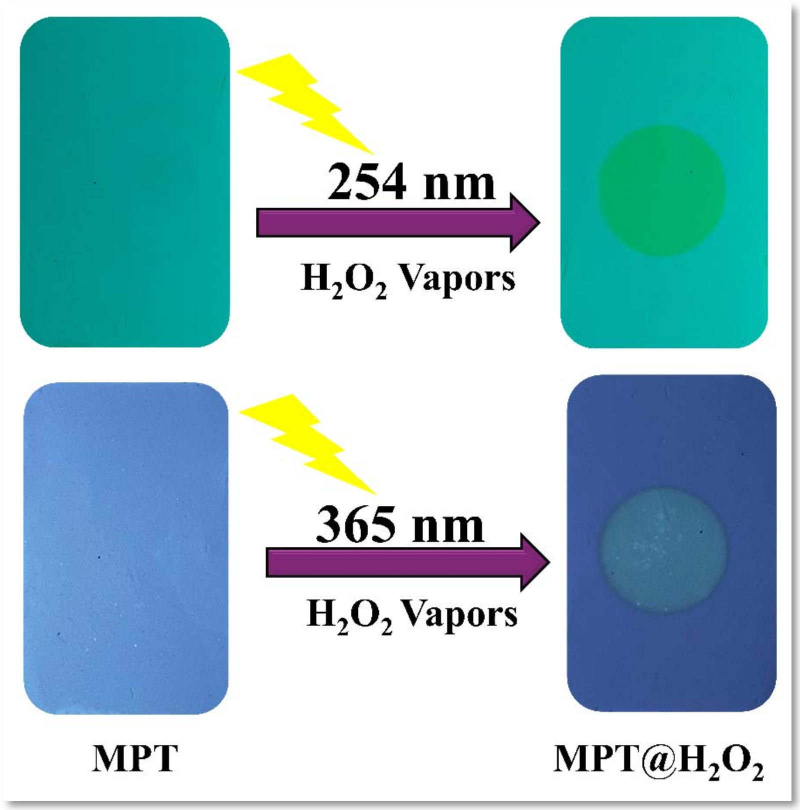

Figure S14: Response of fluorescent paper strip of sensor **MPT** before and after exposure to H_2O_2 vapors.

Table S1. Comparison of **MPT** sensor with already reported H_2O_2 sensor

**Compound**	**LOD (µM)**	**Reference**
MnO_2 nanowires	10	[1]
Fe_3O_4NCs + catalase	0.11	[2]
Au NPs@PB	0.1	[3]
Au nanosheet	1.62	[4]
Cu MOF	4.1	[5]
Chitosan + TMB	1.55	[6]
Pyrimidine based sensor (**MPT**)	**0.08**	**This work**

Table S2. Representation of BCPs of **MPT**@H_2O_2.

**BCPs**	**Probe MPT@ H_2O_2**	**ρ(r) (a.u)**	**Δ^2 ρ(r) (a.u)**	**G(r) (a.u)**	**V(r) (a.u)**	**H(r) (a.u)**	**-V/G**	**Eint (Kcal/mol)**
**61**	H_{11}-O_{32}	0.0234	0.0989	0.0212	-0.0019	0.0035	-0.0896	-0.596
**74**	O_{27}-H_{35}	0.0466	0.2055	0.0507	-0.0501	0.0006	-0.9881	-15.718
**77**	H_{35}-H_{21}	0.0241	0.0774	0.0178	-0.0162	0.0015	-0.9101	-5.082

Table S3. Results for the quantification of hydrogen peroxide in real samples

**Samples**	**Spiked (μM)**	**Recovered (μM)**	**Recovery (%)**	**RSD (%, n =3)**
**Water**	0	Not detected	-	-
	50	49.15	98.3	1.94
	100	98.65	98.65	1.73
**Orange Juice**	0	Not detected	-	-
	50	48.75	97.5	2.32
	100	97.85	97.85	2.07
**Milk**	0	Not detected	-	-
	50	48.55	97.1	1.69
	100	97.3	97.3	2.11

Table S4. Results for the determination of hydrogen peroxide (H_2O_2) in commercially available real samples.

**Samples**	**%v/v**	**Recovered Concentrations (μM)**	**RSD (%, n = 3)**
**Hair Bleach**	0.5	41	1.16
	1	43	1.19
	1.5	88	1.14
	2	109	1.19
**Disinfectant**	0.5	21	1.22
	1	43	1.2
	1.5	87	1.23
	2	131	1.18
**Mouth Wash**	0.5	22	1.23
	1	43	1.18
	1.5	66	1.26
	2	109	1.21

1. Complete reference for Gaussian 09

Gaussian 09, Revision D.01,M. J. Frisch, G. W. Trucks, H. B. Schlegel, G. E. Scuseria, M. A. Robb, J. R. Cheeseman, G. Scalmani, V. Barone, B. Mennucci, G. A. Petersson, H. Nakatsuji, M. Caricato, X. Li, H. P. Hratchian, A. F. Izmaylov, J. Bloino, G. Zheng, J. L. Sonnenberg, M. Hada, M. Ehara, K. Toyota, R. Fukuda, J. Hasegawa, M. Ishida, T. Nakajima, Y. Honda, O. Kitao, H. Nakai, T. Vreven, J. A. Montgomery, Jr., J. E. Peralta, F. Ogliaro, M. Bearpark, J. J. Heyd, E. Brothers, K. N. Kudin, V. N. Staroverov, T. Keith, R. Kobayashi, J. Normand, K. Raghavachari, A. Rendell, J. C. Burant, S. S. Iyengar, J. Tomasi, M. Cossi, N. Rega,J. M. Millam, M. Klene, J. E. Knox, J. B. Cross, V. Bakken, C. Adamo, J. Jaramillo,R. Gomperts, R. E. Stratmann, O. Yazyev, A. J. Austin, R. Cammi, C. Pomelli, J. W. Ochterski, R. L. Martin, K. Morokuma,V. G. Zakrzewski, G. A. Voth, P. Salvador, J. J. Dannenberg, S. Dapprich, A. D. Daniels, O. Farkas, J. B. Foresman, J. V. Ortiz, J. Cioslowski, and D. J. Fox, Gaussian, Inc., Wallingford CT, 2013.

References

[1] Dong, S., Xi, J., Wu, Y., Liu, H., Fu, C., Liu, H., et al. High loading MnO2 nanowires on graphene paper: facile electrochemical synthesis and use as flexible electrode for tracking hydrogen peroxide secretion in live cells. Analytica Chimica Acta 2015, 853, 200-6.

[2] Kıranşan, K.D., Aksoy, M., Topçu, E. Flexible and freestanding catalase-Fe3O4/reduced graphene oxide paper: Enzymatic hydrogen peroxide sensor applications. Materials Research Bulletin 2018, 106, 57-65.

[3] Zhang, M., Halder, A., Hou, C., Ulstrup, J., Chi, Q. Free-standing and flexible graphene papers as disposable non-enzymatic electrochemical sensors. Bioelectrochemistry 2016, 109, 87-94.

[4] Peng, Y., Lin, D., Gooding, J.J., Xue, Y., Dai, L. Flexible fiber-shaped non-enzymatic sensors with a graphene-metal heterostructure based on graphene fibres decorated with gold nanosheets. Carbon 2018, 136, 329-36.

[5] Ling, W., Hao, Y., Wang, H., Xu, H., Huang, X. A novel Cu-metal-organic framework with two-dimensional layered topology for electrochemical detection using flexible sensors. Nanotechnology 2019, 30, 424002.

[6] Ragavan, K., Ahmed, S.R., Weng, X., Neethirajan, S. Chitosan as a peroxidase mimic: Paper based sensor for the detection of hydrogen peroxide. Sensors and Actuators B: Chemical 2018, 272, 8-13.

SI-2. NMR Spectra of Sensor MPT

^{1}H-NMR of MPT (DMSO-d_6, 400 MHz)

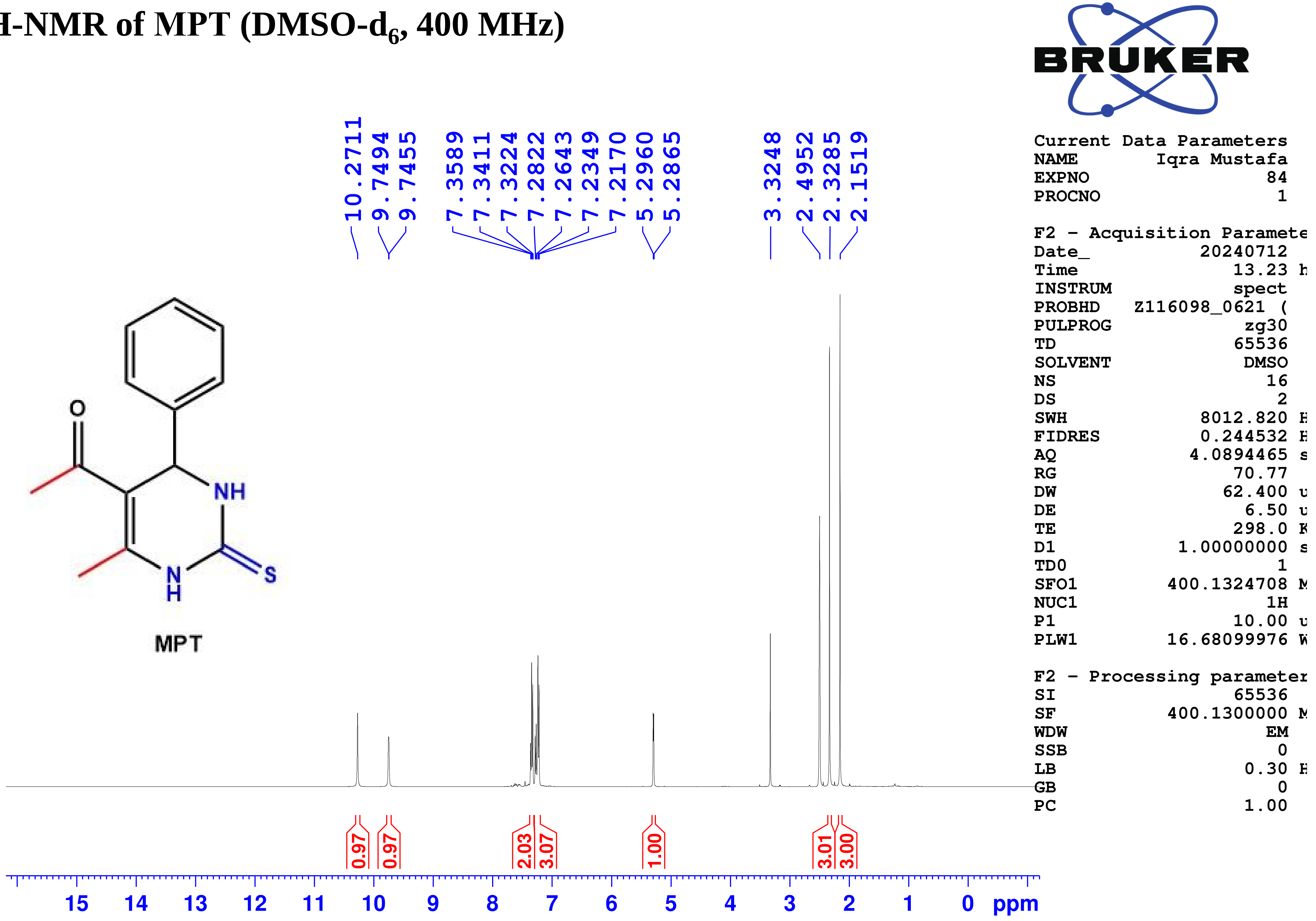

^{1}H-NMR of MPT (DMSO-d_6, 400 MHz)

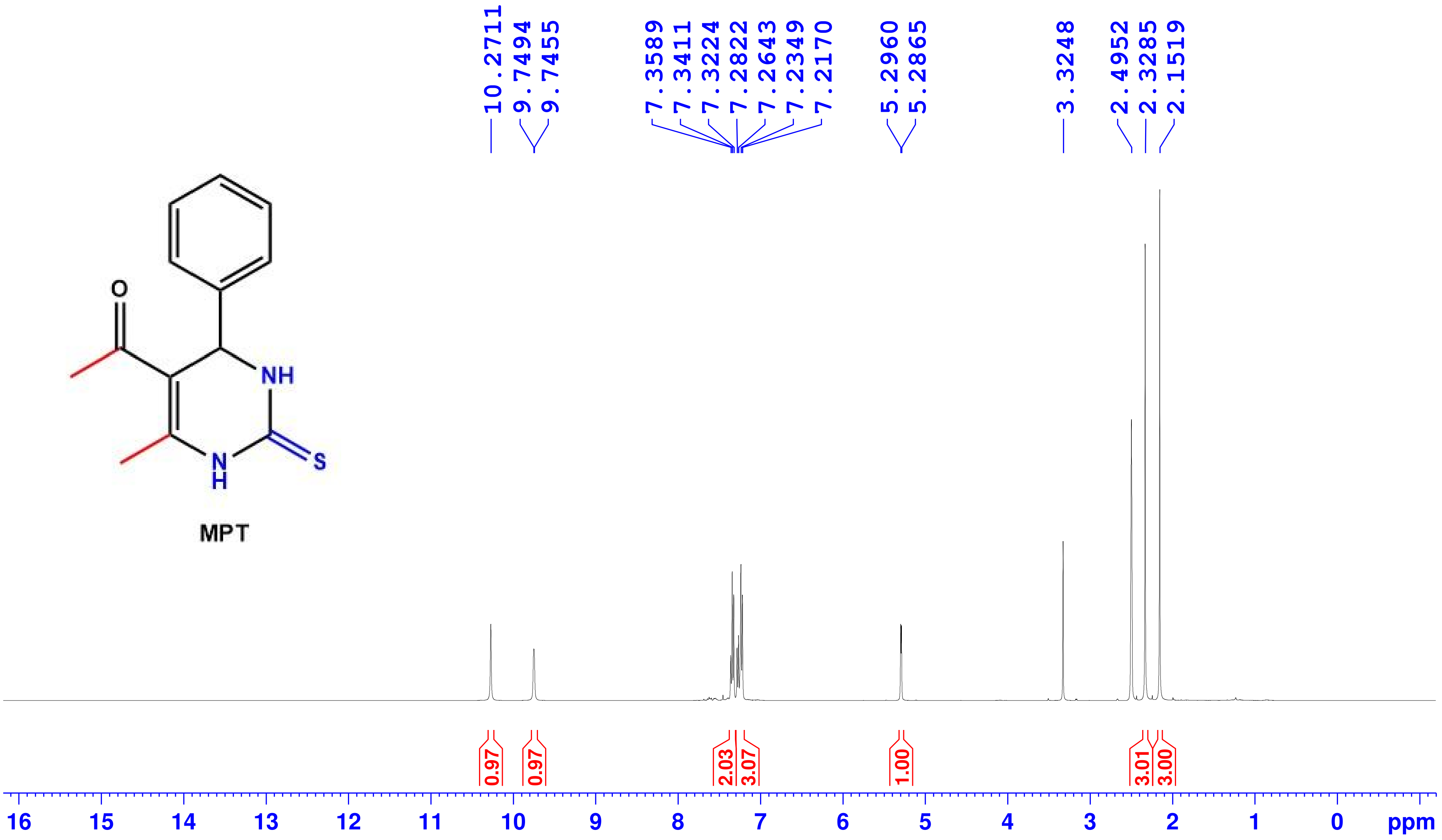

^{1}H-NMR of MPT (DMSO-d_6, 400 MHz)

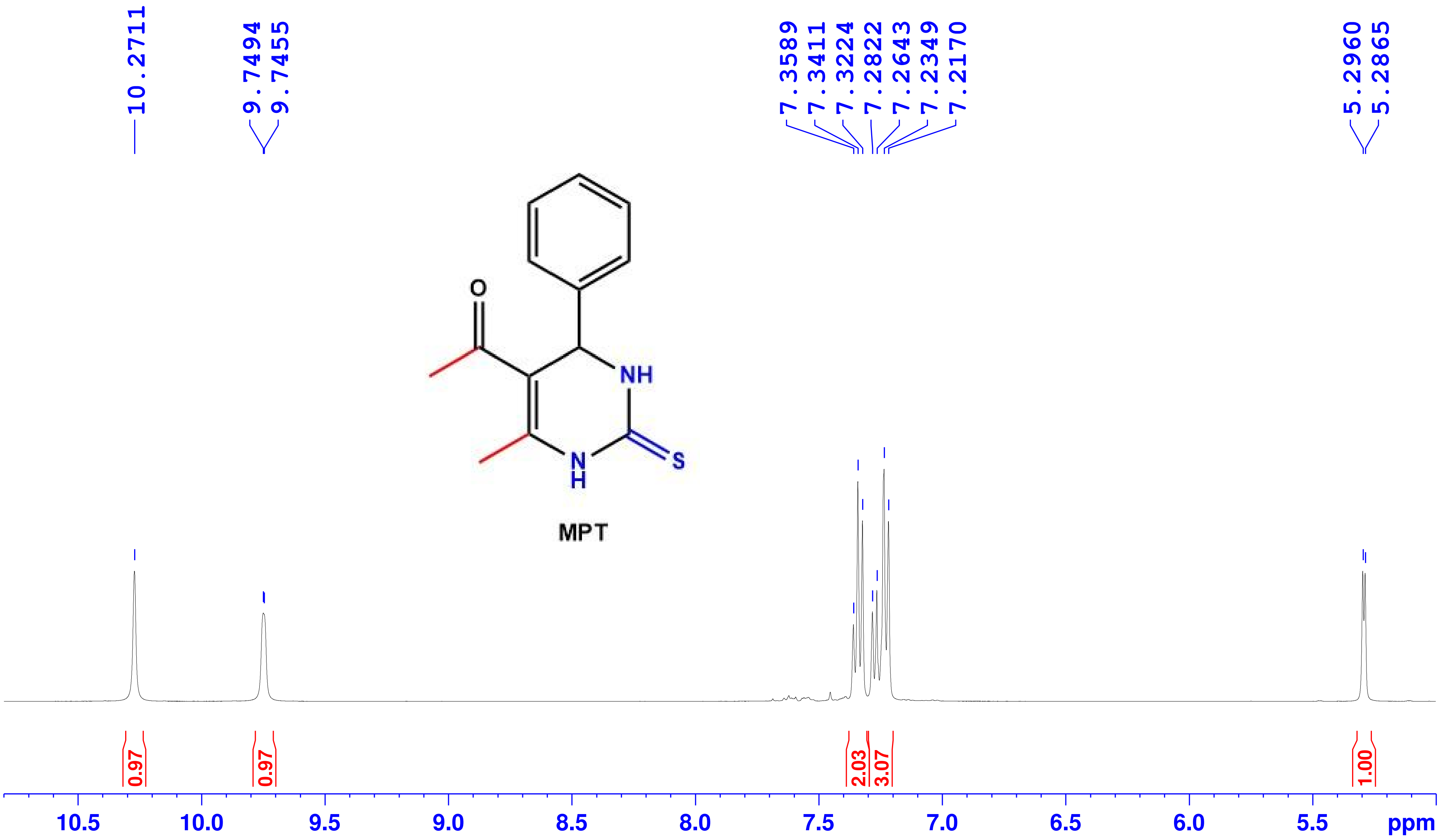

^{1}H-NMR of MPT (DMSO-d_6, 400 MHz)

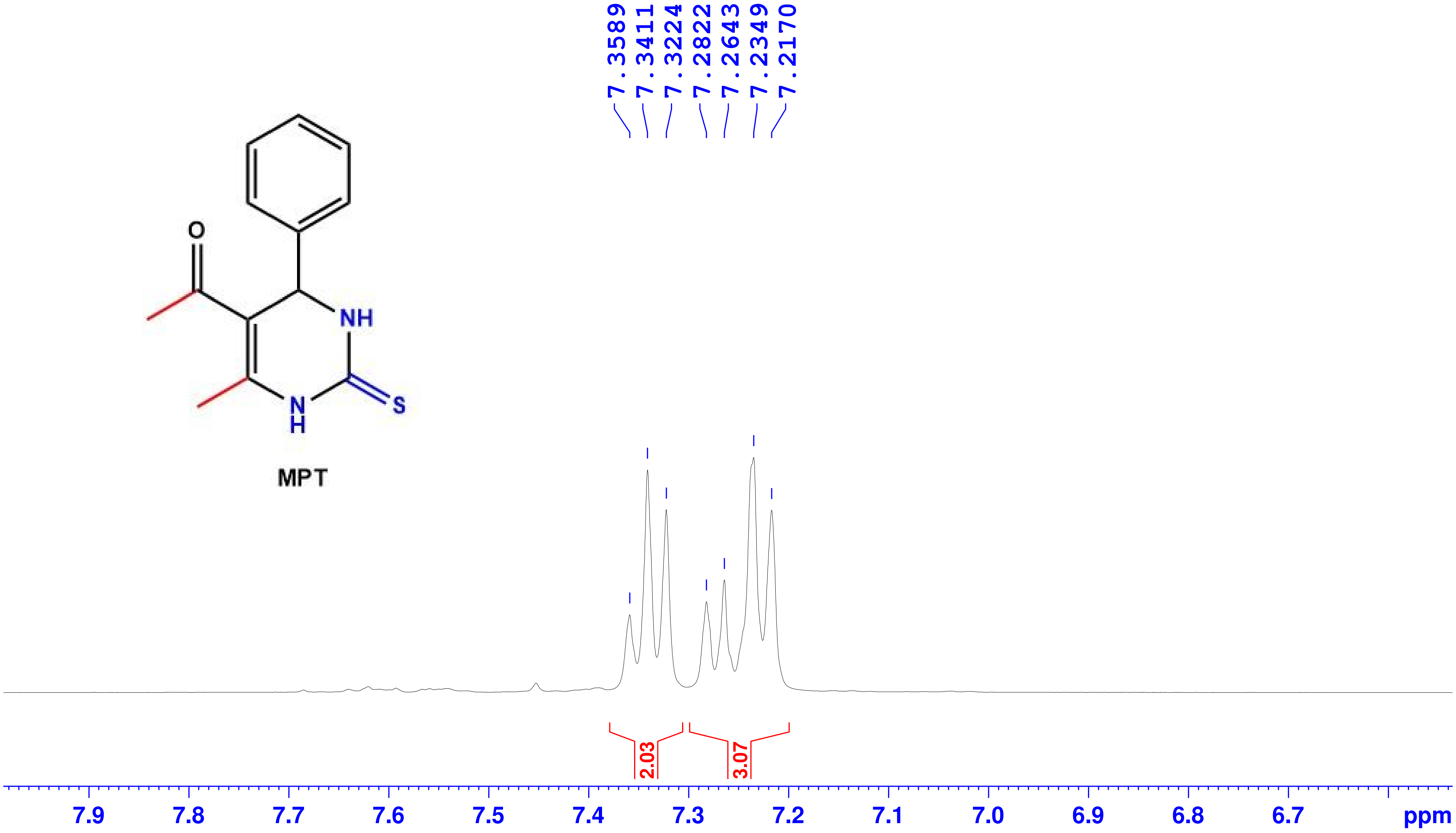

^{1}H-NMR of MPT (DMSO-d_6, 400 MHz)

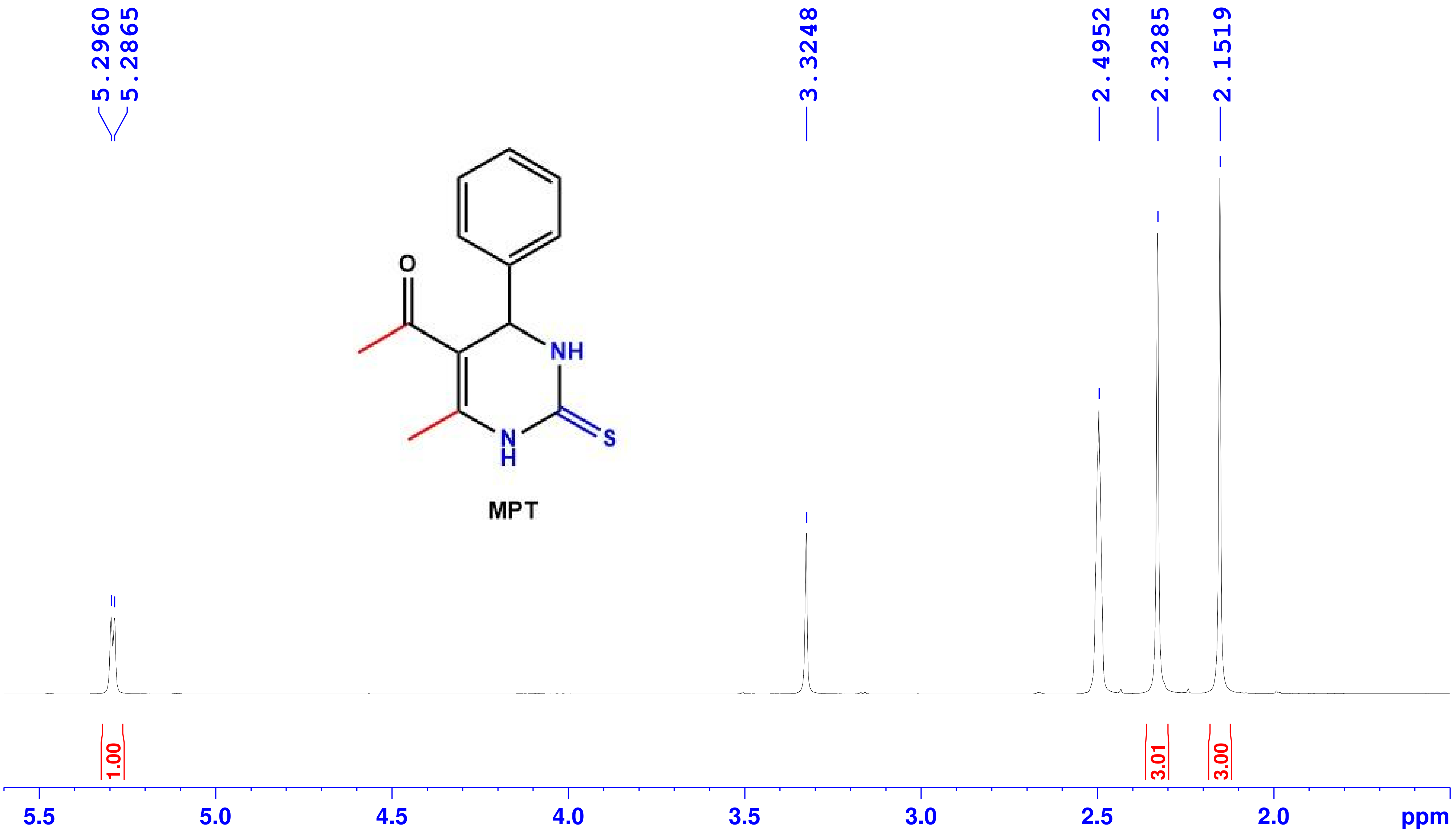

^{13}C-NMR of MPT (DMSO-d_6, 100 MHz)

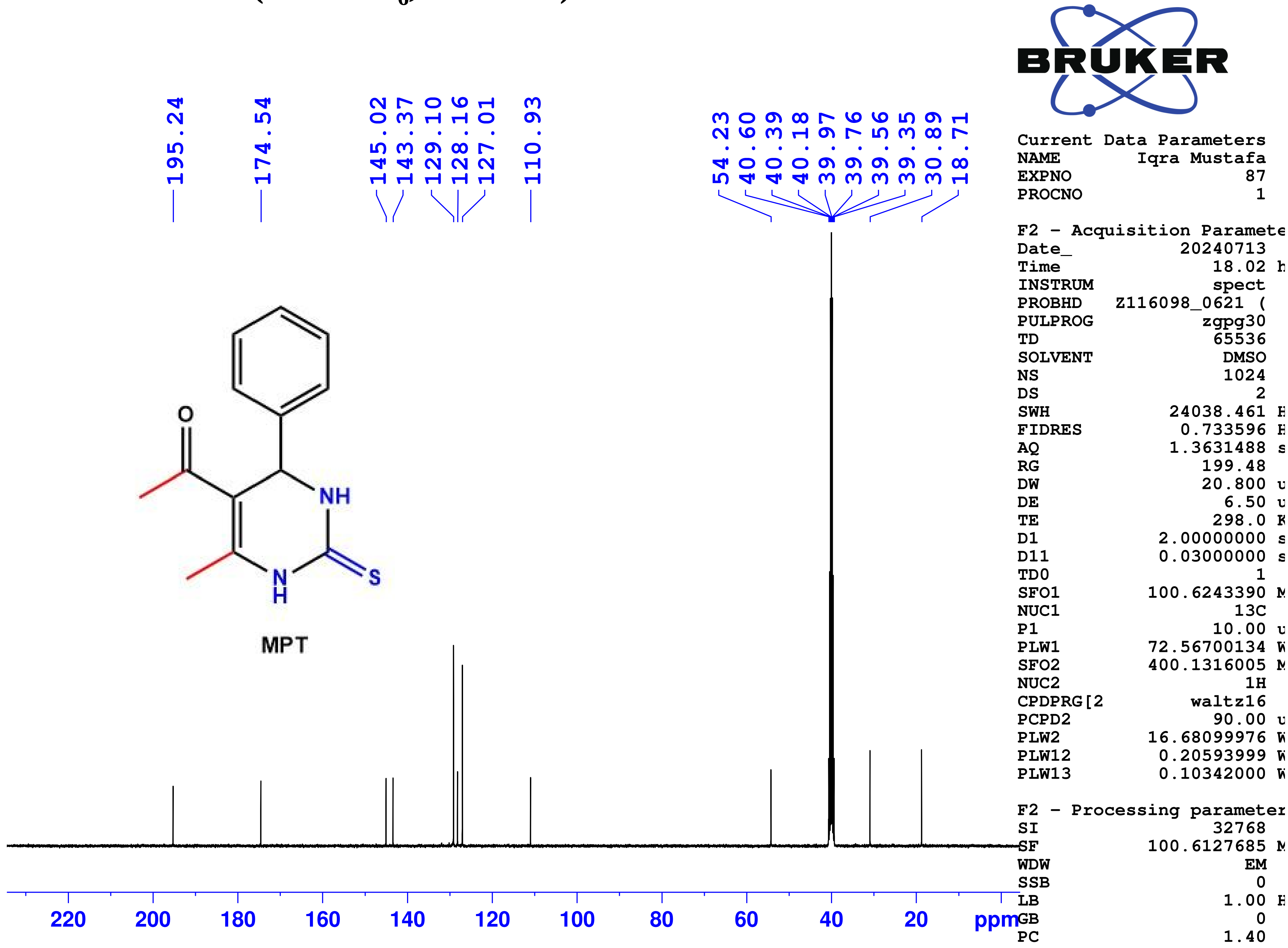

^{13}C-NMR of MPT (DMSO-d_6, 100 MHz)

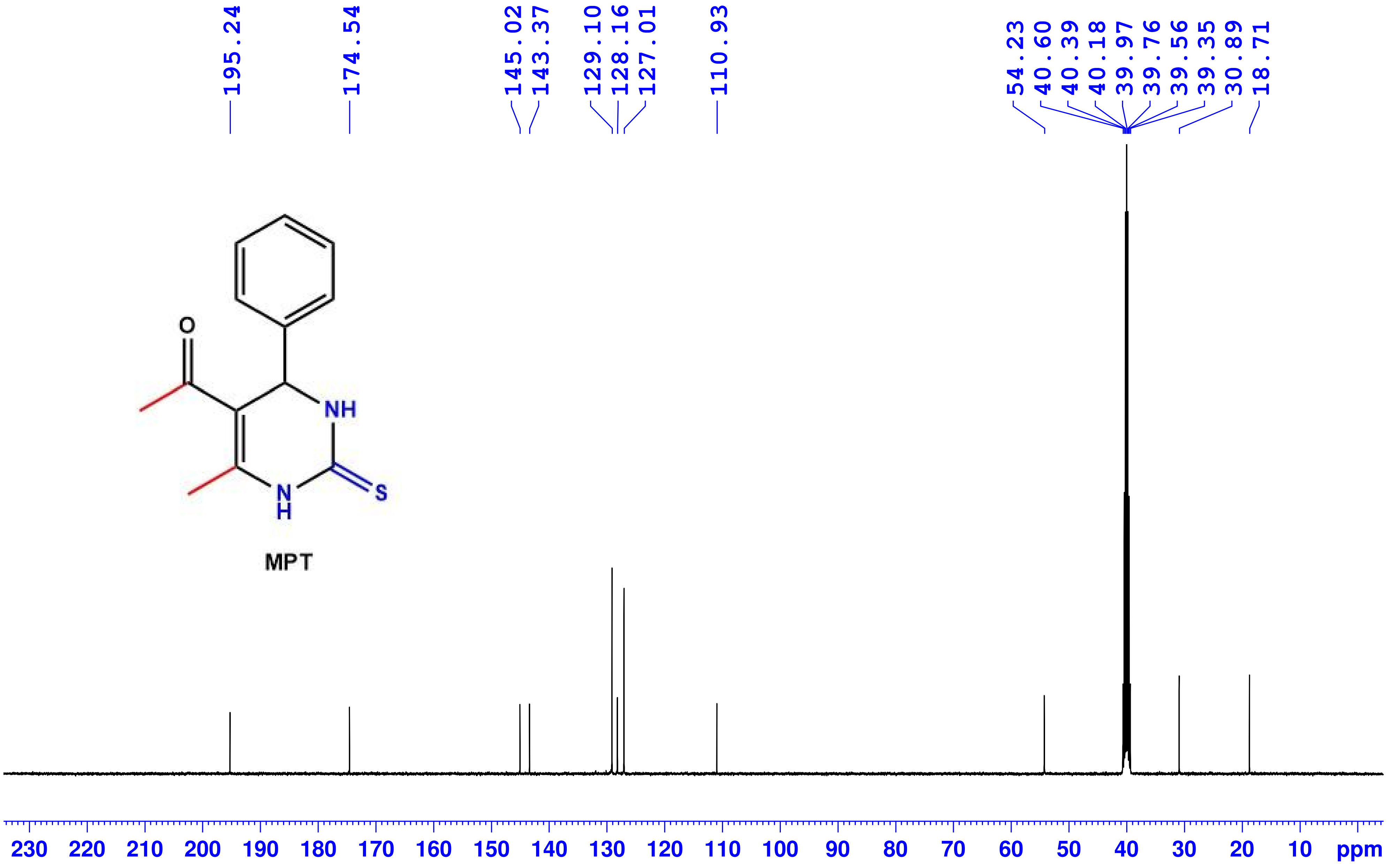

^{13}C-NMR of MPT (DMSO-d_6, 100 MHz)

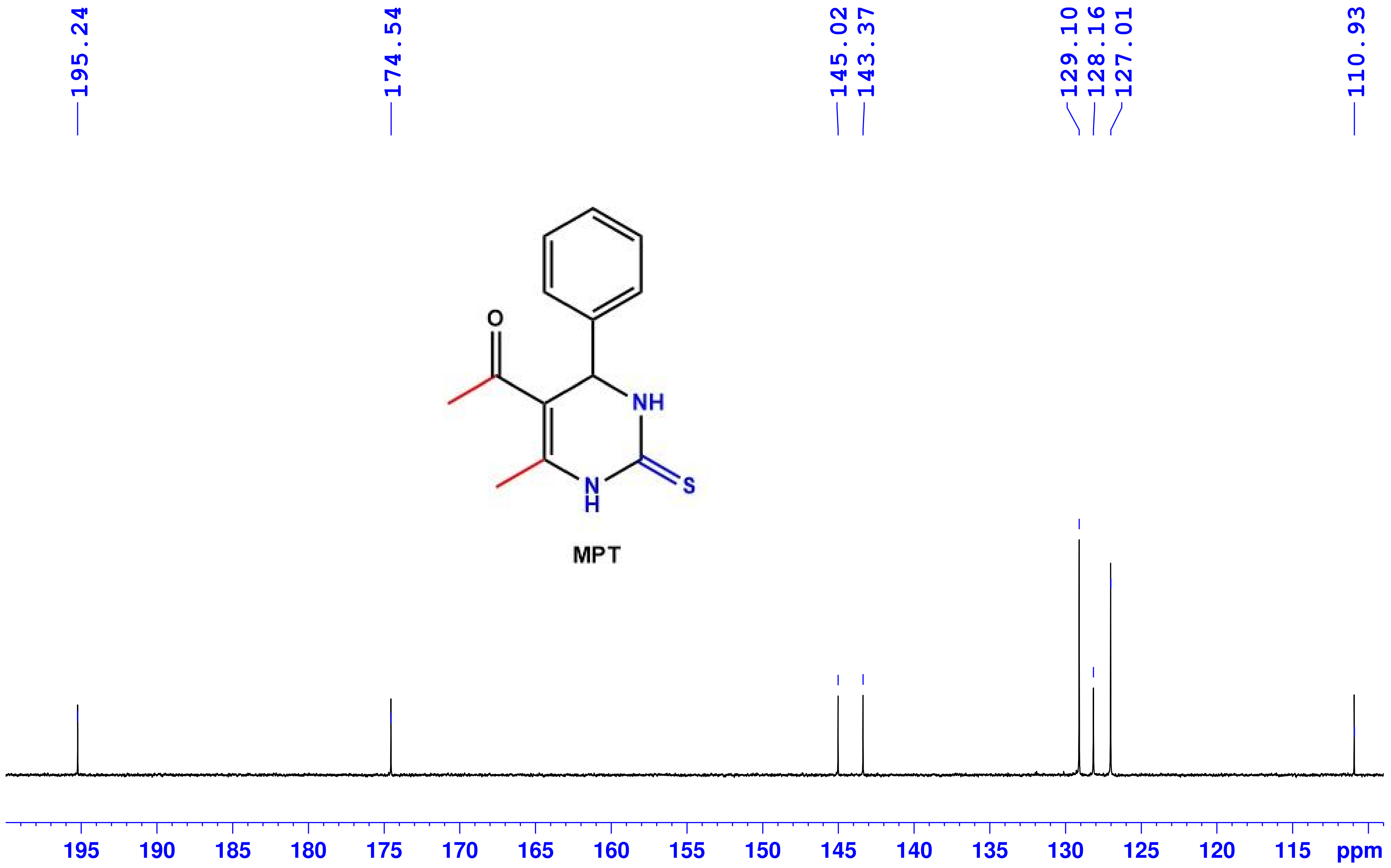

^{13}C-NMR of MPT (DMSO-d_6, 100 MHz)

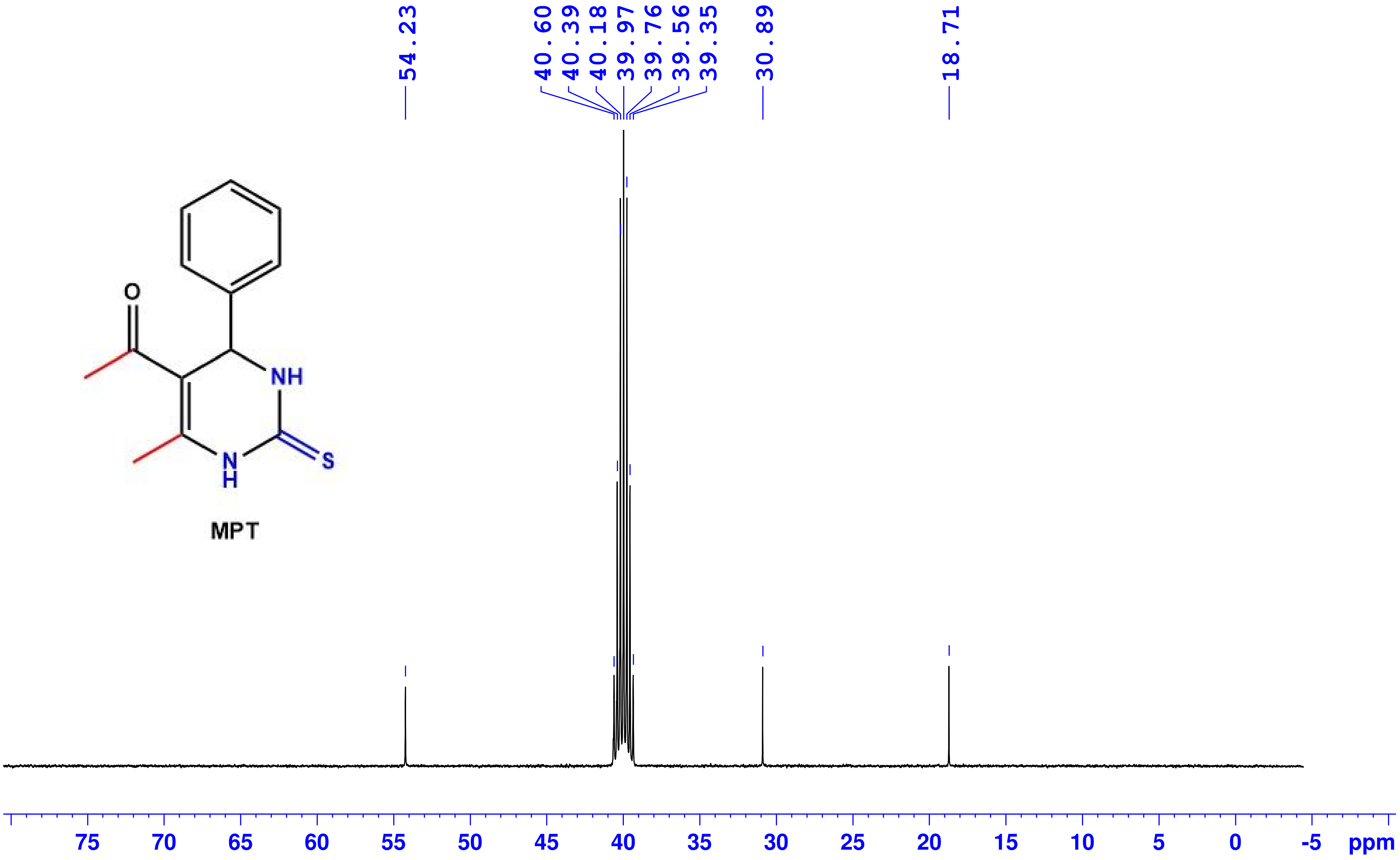

DEPT-135 of MPT (DMSO-d_6, 100 MHz)

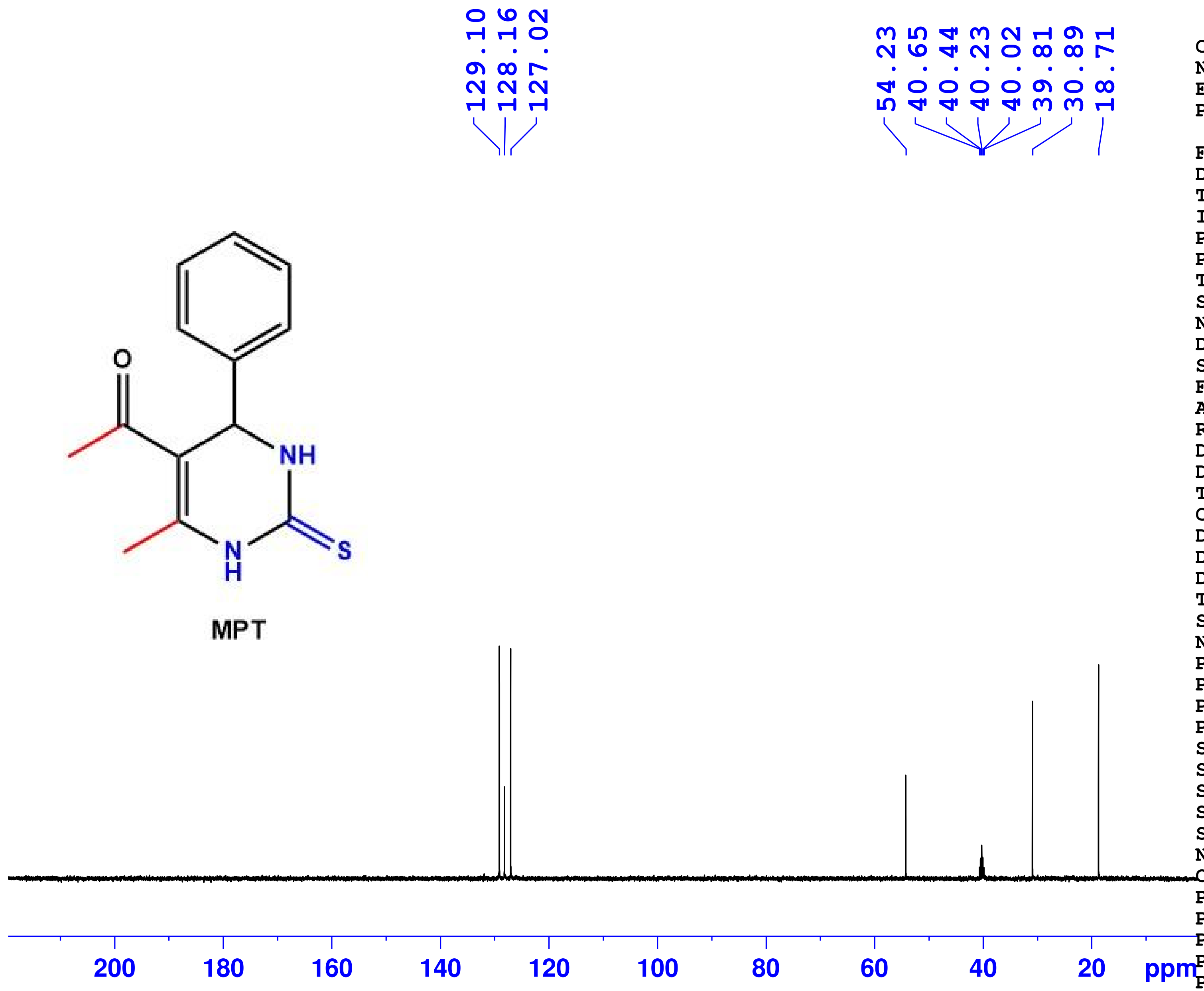

Current Data Parameters

NAME	Iqra Mustafa
EXPNO	88
PROCNO	1

F2 - Acquisition Paramete

Date_	20240713
Time	18.18 h
INSTRUM	spect
PROBHD	Z116098_0621 (
PULPROG	deptsp135
TD	65536
SOLVENT	DMSO
NS	256
DS	4
SWH	22058.824 H
FIDRES	0.673182 H
AQ	1.4854827 s
RG	199.48
DW	22.667 u
DE	6.50 u
TE	298.0 K
CNST2	145.0000000
D1	2.00000000 s
D2	0.00344828 s
D12	0.00002000 s
TD0	1
SFO1	100.6238359 M
NUC1	13C
P1	10.00 u
P13	2000.00 u
PLW0	0 W
PLW1	72.56700134 W
SPNAM[5]	Crp60comp.4
SPOAL5	0.500
SPOFFS5	0 H
SPW5	11.08699989 W
SFO2	400.1316005 M
NUC2	1H
CPDPRG[2	waltz16
P3	10.00 u
P4	20.00 u
PCPD2	90.00 u
PLW2	16.68099976 W
PLW12	0.20593999 W

DEPT-135 of MPT (DMSO-d_6, 100 MHz)

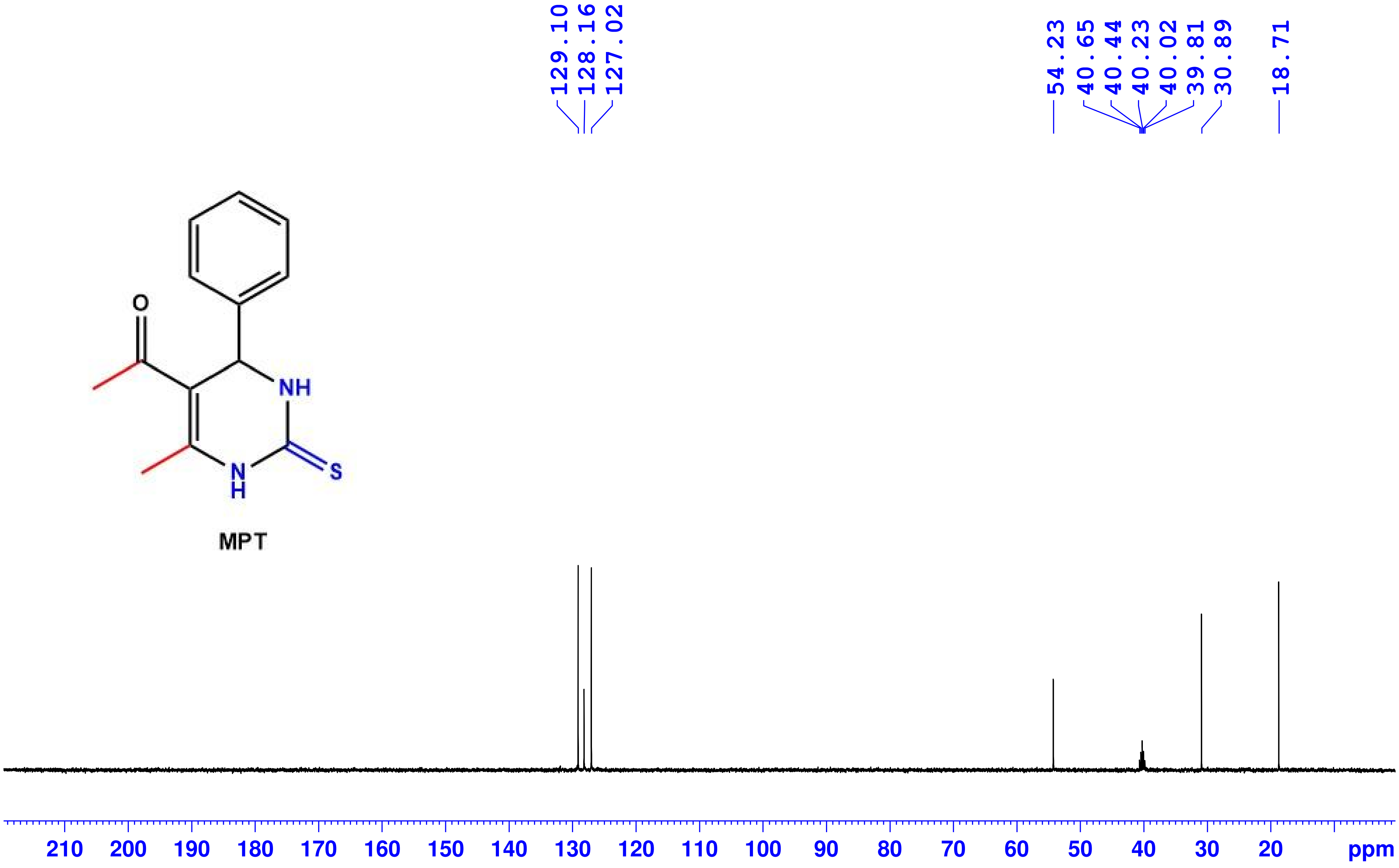

DEPT-135 of MPT (DMSO-d_6, 100 MHz)

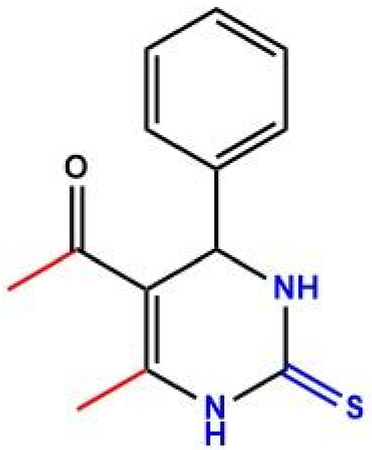

MPT

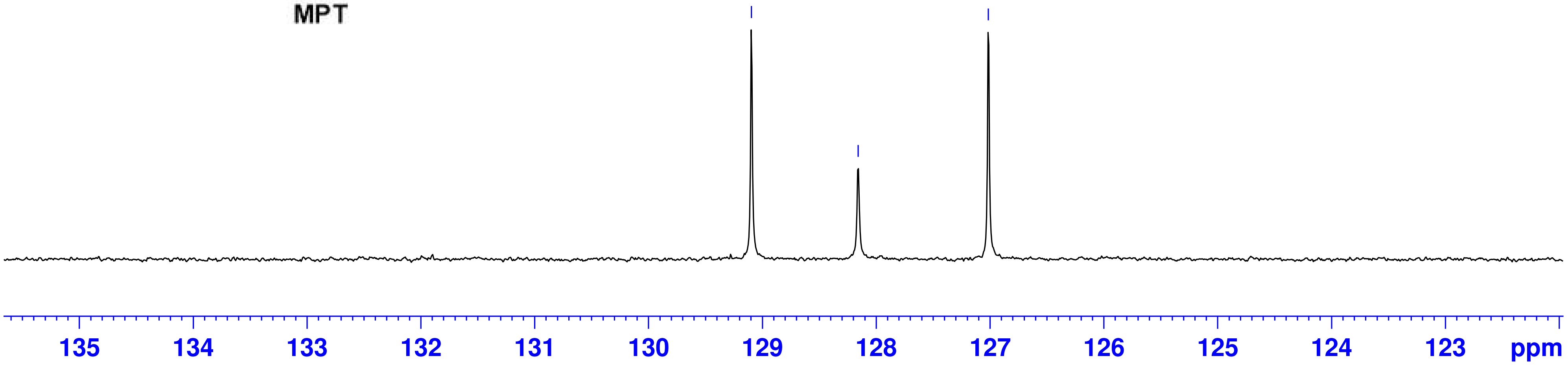

DEPT-135 of MPT (DMSO-d_6, 100 MHz)

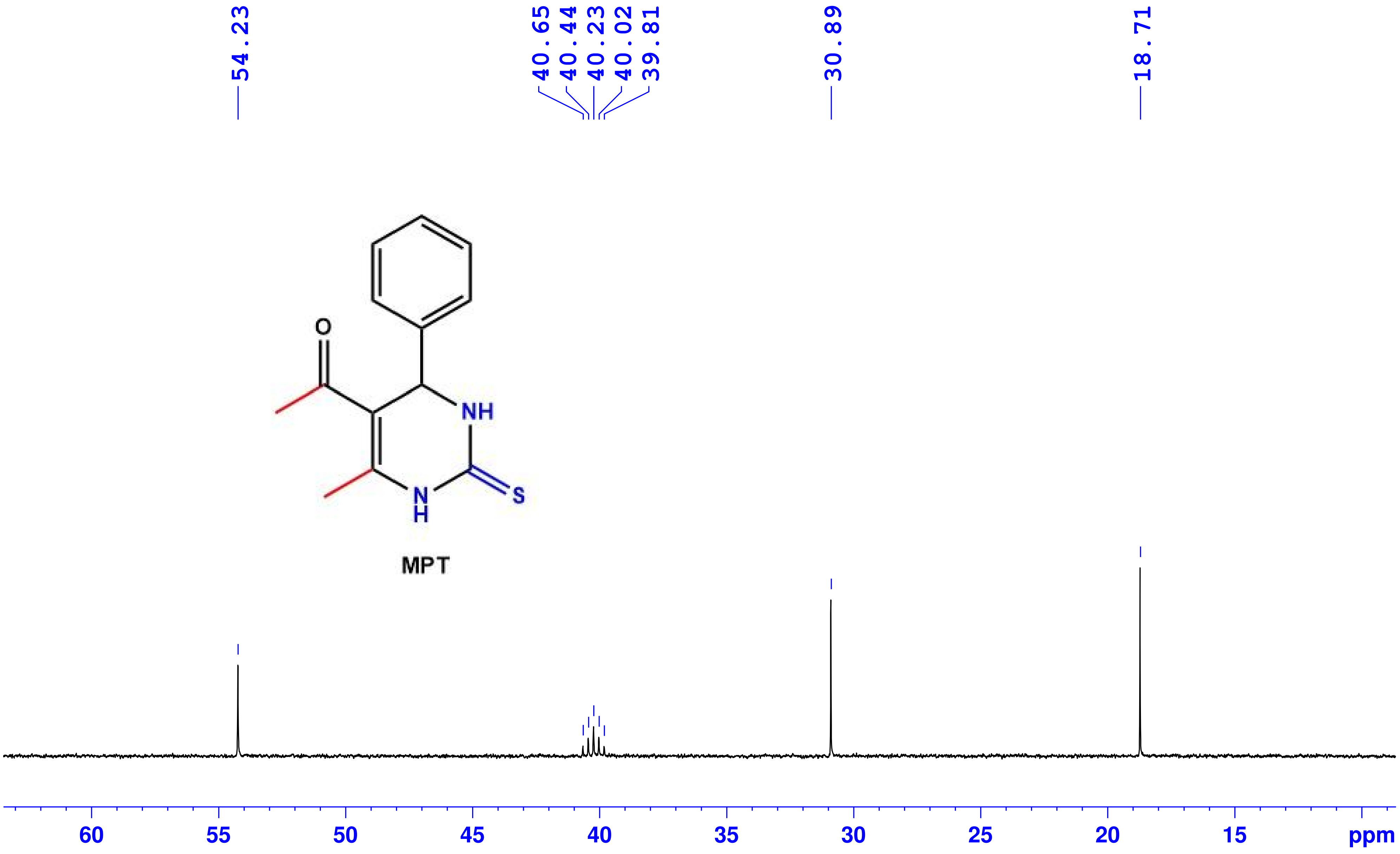

^{1}H-NMR of MPT with 4-NA (DMSO-d_6, 400 MHz)

Titration NMR

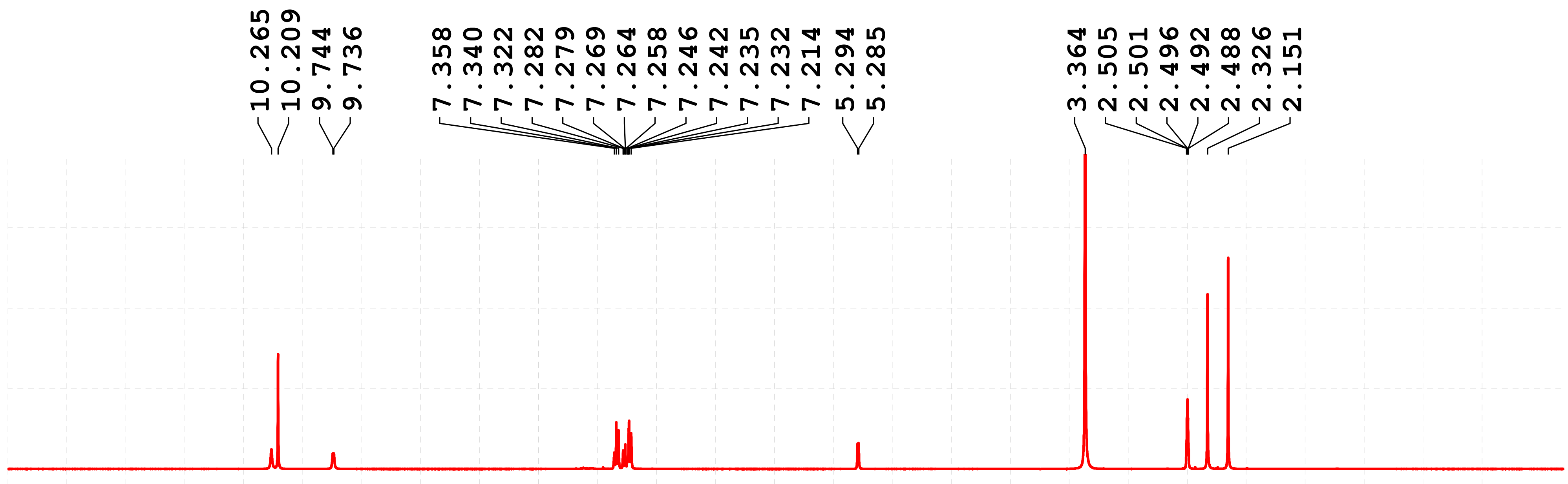

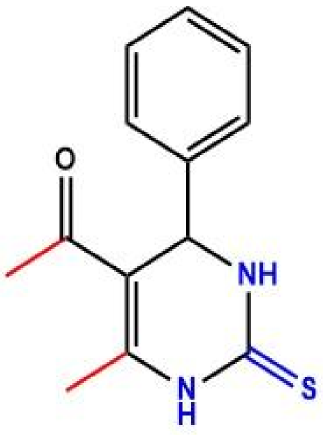

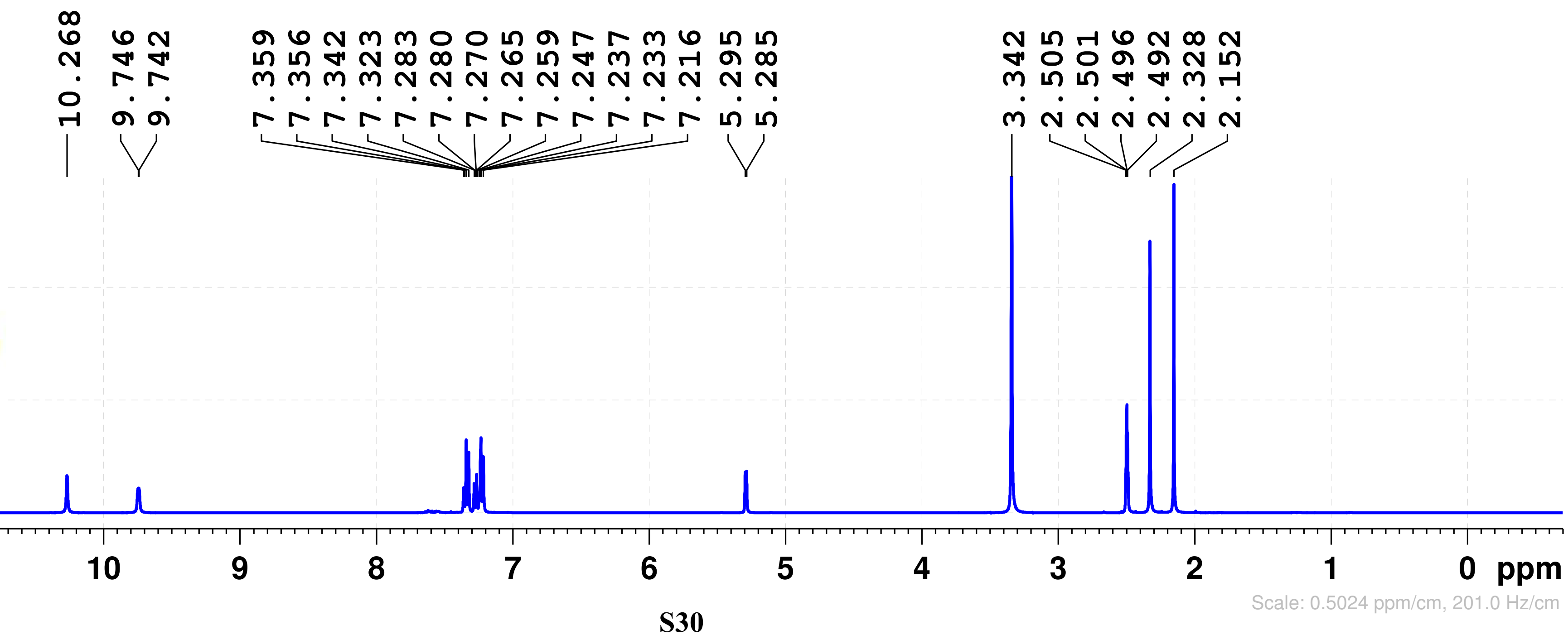

^{1}H-NMR of MPT with 4-NA (DMSO-d_6, 400 MHz)

Titration NMR

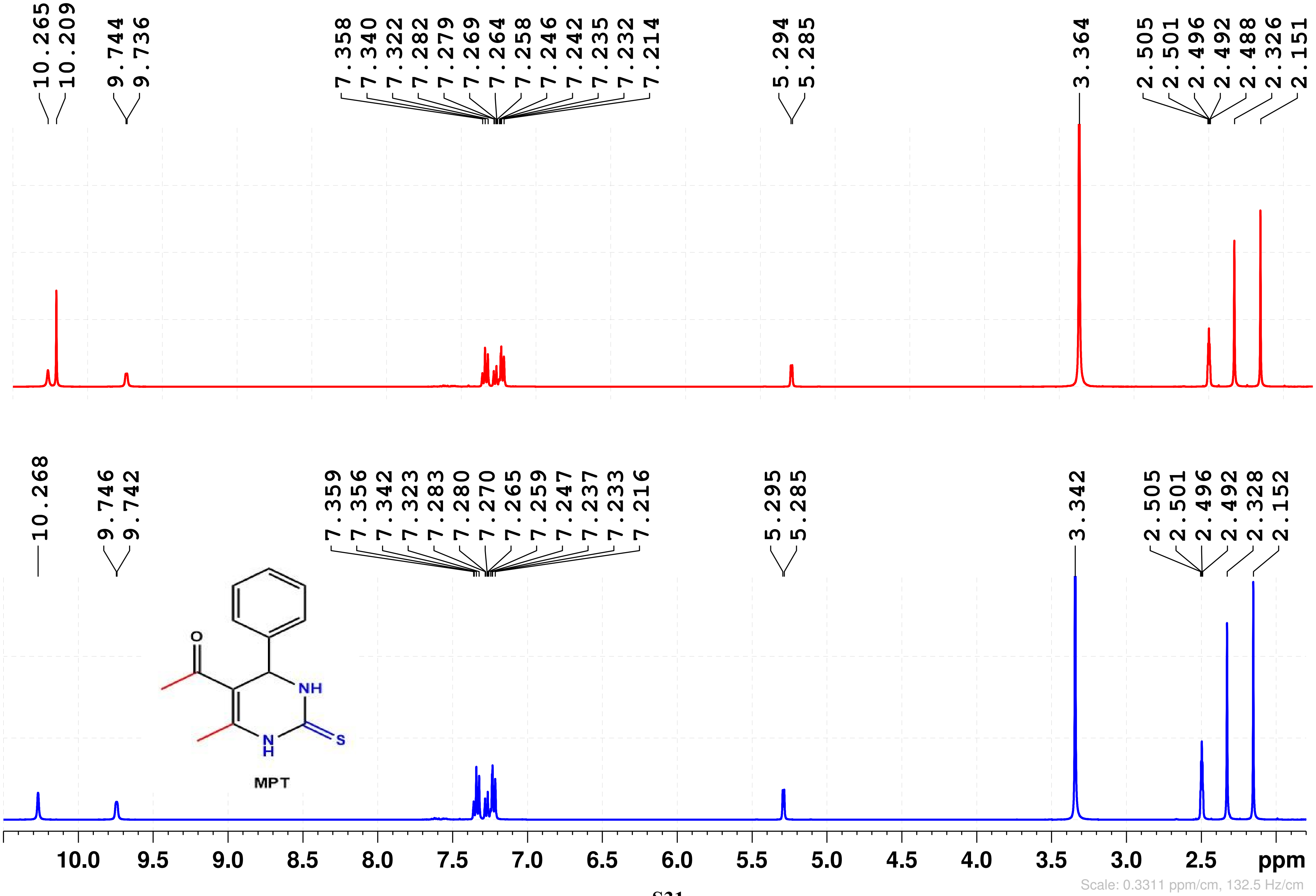

^{1}H-NMR of MPT with 4-NA (DMSO-d_6, 400 MHz)

Titration NMR

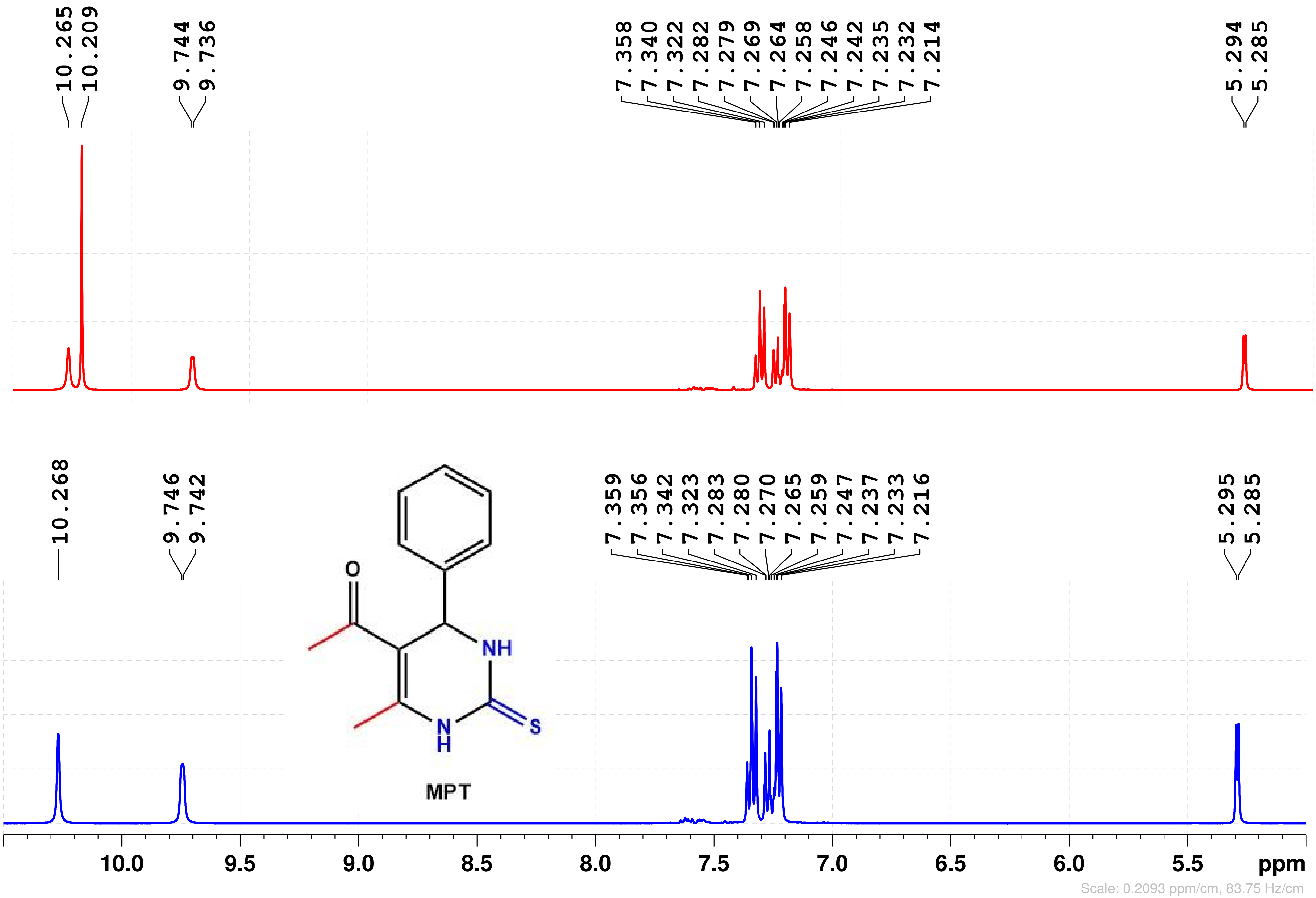

^{1}H-NMR of MPT with 4-NA (DMSO-d_6, 400 MHz) Titration NMR

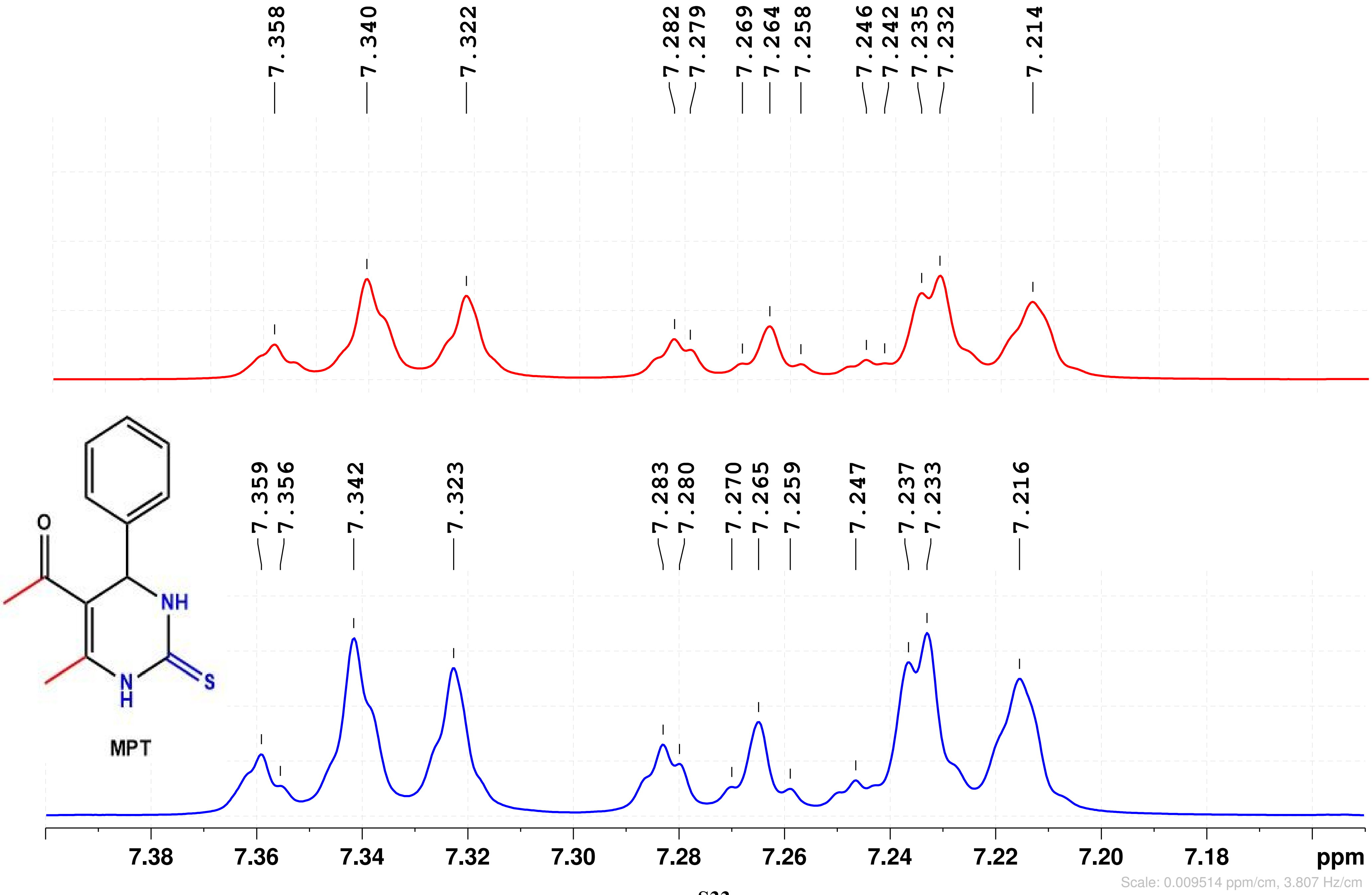

Supplement: RA-016-D6RA02128J-s001 [file RA-016-D6RA02128J-s001.pdf]
